# Effect of Physical Activity on Cognitive Impairment in Patients With Cerebrovascular Diseases: A Systematic Review and Meta-Analysis

**DOI:** 10.3389/fneur.2022.854158

**Published:** 2022-05-06

**Authors:** Huawei Lin, HuanHuan Liu, Yaling Dai, Xiaolong Yin, Zuanfang Li, Lei Yang, Jing Tao, Weilin Liu, Lidian Chen

**Affiliations:** ^1^College of Rehabilitation Medicine, Fujian University of Traditional Chinese Medicine, Fuzhou, China; ^2^National-Local Joint Engineering Research Center of Rehabilitation Medicine Technology, Fujian University of Traditional Chinese Medicine, Fuzhou, China; ^3^The Academy of Rehabilitation Industry, Fujian University of Traditional Chinese Medicine, Fuzhou, China

**Keywords:** cerebrovascular disease, cognitive function, meta-analysis, physical activity, stroke, systematic review, vascular cognitive impairment

## Abstract

**Background and Purpose:**

This study investigates the effect of physical activity (PA) on cognition in patients with cerebrovascular disease and explored the maximum benefit of different PA characteristics.

**Methods:**

Databases, such as Pubmed, Web of Science, Embase, and Cochrane Library, were searched from their inception to May 31, 2021. Standardized mean difference (SMD) and 95% confidence intervals (*CI*s) were calculated to generate a forest plot. In addition, subgroup analysis, moderation analysis, and regression analysis were performed to explore the possible adjustment factors.

**Results:**

In total, 22 studies that met the criteria were included, demonstrating data from 1,601 participants. The results indicated that PA produced a positive effect on the global cognition for patients with cerebrovascular disease (SMD: 0.20 [95% *CI*: 0.12–0.27]), at the same time, PA training prominently improved executive function (SMD: 0.09 [95% *CI*: 0.00–0.17]) and working memory (SMD: 0.25 [95% *CI*: 0.10–0.40]). Furthermore, patients with baseline cognitive impairment received the greater benefit of PA on cognition (SMD: 0.24 [95% *CI*: 0.14–0.34]) than those without cognitive impairment before intervention (SMD: 0.15 [95% *CI*: 0.04–0.26]). For patients in the acute stage (≤ 3 months), PA did not rescue impairment dysfunction significantly (SMD: 0.08 [95% *CI*: −0.04–0.21]) and remarkable cognitive gains were detected in the chronic stage of participants (>3 months) (SMD: 0.25 [95% *CI*: 0.16–0.35]). Moderate intensity PA showed a larger pooled effect size (SMD: 0.23 [95% *CI*: 0.11–0.36]) than low intensity (SMD: −0.01 [95% *CI*: −0.44–0.43]) and high intensity (SMD: 0.16 [95% *CI*: 0.03–0.29]). However, the different types, duration, and frequency of PA resulted in no differences in the improvement of cognitive function. Further regression analysis demonstrated that the beneficial effects of PA on cognition are negatively correlated with age (*p* < 0.05).

**Conclusions:**

This study revealed that PA can prominently improve the cognitive ability in patients with cerebrovascular diseases and strengthened the evidence that PA held promise as a widely accessible and effective non-drug therapy for vascular cognitive impairment (VCI).

## Introduction

Cerebrovascular disease is a type of disease that causes disorders of intracranial blood circulation and damage to the brain tissue. Clinically, it is manifested as severe motor and cognitive dysfunction, which brings a great burden to the patient's family and social medical system. Stroke is the most prevalent in cerebrovascular diseases. Studies have indicated that over 50% of stroke survivors developed a degree of cognitive impairment (1, 2). In addition, chronic hypoperfusion cerebral ischemia, mainly due to cerebral small vessel diseases, is also a major cause of vascular cognitive impairment (VCI) (3). A growing number of studies have found that cognitive impairment was one of the main complication after stroke. According to reports, stroke increased the risk of cognitive impairment by at least 5–8 times compared with age-matched controls, who were >60 years of age (4, 5). A study suggested that ~83% of stroke survivors showed impairment at least one cognitive domain, and cognitive impairment was prevalent in patients with successful clinical recovery (6). Therefore, the cognitive function has become one of the main research objects and outcome indicators in cerebrovascular diseases. Given the current lack of effective clinical drug treatments, identifying and promoting safe and effective alternative treatments are imperative.

For a long time, physical activity (PA) has been widely accepted as an effective and low-cost treatment for balance function and limb dyskinesia after stroke (7, 8). Randomized controlled trials have shown that PA training can effectively improve the overall cognitive function of stroke patients (9). Moreover, long-term regular aerobic exercise for subjects with mild subcortical ischemic significantly reduced the scores of the Alzheimer's Disease Assessment Scale-Cognitive Scale (ADAS-cog), indicating positive cognitive gains (10). However, some articles have put forward a different point of view, whose findings suggested that there was no statistically significant correlation between PA training and the beneficial influence on cognitive function after stroke (11, 12). As far as the current evidence is concerned, the specific effect of PA training on overall cognitive function is still inconclusive. A meta-analysis of 14 studies published in 2017 indicated that PA training exerted a significant and positive impact on cognition and further explored the research and sample characteristics related to the efficacy of PA (13). Nevertheless, in the past 5–6 years, the number of trials investigating the effect of PA training on improving VCI has grown rapidly, including some studies with negative results (11, 14, 15), so it is warranted to renew such an updated quantitative synthesis involving the latest trails.

Therefore, we conducted the latest meta-analysis and systematic review. Compared with the past, we first expanded the scope of inclusion of the population. Then, through moderation analysis and regression analysis, we explored the factors that may affect the efficacy of PA on cognitive performance from a broader perspective, such as the type of control, cognitive status before the intervention, age, gender, stroke type, hemiplegic side, and frequency of PA, which have not been involved in previous studies. Finally, in the research of domain specificity, we have not only investigated the effects of PA on executive function and working memory. At the same time, the data analysis of PA training on spatial function has been added, although there are few types of research in this part at present. In general, this study aimed to clarify the impact of PA on the cognitive function of patients with cerebrovascular disorders and the potential influencing factors, providing a theoretical basis for the choice of treatment timing and the formulation of exercise programs.

## Methods

The meta-analysis was conducted in accordance with the guidelines of the Preferred Reporting Items for Systematic Reviews and Meta-analysis (PRISMA) statement and was registered at the International Prospective Register of Systematic Reviews (https://www.crd. york.ac.uk/PROSPERO/) (number CRD42021258351).

### Search Strategy

Two investigators independently performed a literature search in the databases of Pubmed, Web of Science, Embase, and Cochrane Library from the earliest available record up to May 31, 2021. Besides, we manually searched the reference lists of related articles. The keywords and detailed search terms were as following: ((((((((((((physical activity) OR (training)) OR (exercise)) OR (aerobic)) OR (mind-body)) OR (endurance)) OR (resistance)) OR (strength)) OR (fitness)) OR (physiotherapy)) AND ((((((((cerebral hemorrhage) OR (cerebral infarction)) OR (cerebral ischemia)) OR (stroke)) OR (post-stroke)) OR (vascular dementia)) OR (cerebrovascular accident)) OR (transient ischemia attack))) AND (((((((((((cognition) OR (cognitive function)) OR (vascular cognition impairment)) OR (processing)) OR (language)) OR (visuospatial)) OR (attention)) OR (memory)) OR (executive function)) OR (intelligence)) OR (neuropsychological))) AND (randomized controlled trial).

### Study Selection

Studies were included if they met the following criteria: (1) subjects with the cerebrovascular disorder were confirmed by clinical evidence and ≥18 years old; (2) included studies must define a control group, whose intervention did not contain PA components or can be allowed to contain the conventional exercise components (stretching, balance exercise, and physiotherapy). In addition, if the control group included PA components, but a certain type of exercise was the only difference in intervention between groups, it can be included; (3) the duration of PA lasted for at least 4 weeks; (4) at least one recognized global cognitive function neuropsychological test or multiple neuropsychological tests that evaluated across multiple domains must be included, and data before and after the intervention or the data of change from pre- to postintervention can be obtained; (5) we have no restrictions on the types of cerebrovascular diseases and whether there were cognitive impairments before intervention; however, included subjects cannot have other neurodegenerative diseases or serious mental diseases that can cause cognitive impairment; and (6) randomized controlled trial.

### Data Collection and Extraction

Two individuals independently extracted the original data from the published article or the table in the supplementary materials and the data were checked by a third researcher. The extracted details included publication year and country, participant characteristic (age, gender and sample size, education, and body mass index), stroke characteristic (stroke duration, site of the stroke, and stroke type), intervention characteristics (type of PA, intensity, duration, and frequency). In addition, the data of cognitive status before the intervention, cognitive outcomes, cognitive domains, and type of measurement were also extracted. For trials that included post-intervention and follow-up data, only data immediately after the intervention were extracted. According to an intent-to-treat framework, sample sizes at baseline, rather than post-intervention, were adopted to calculate the pooled effect size. The effect of PA on the overall cognitive function was regarded as the primary outcome, while the specific moderating effects of different influencing factors for PA on the improvement of cognition and the impact of PA on specific cognitive domains were considered as the secondary outcome.

### Quality Assessment

Two researchers assessed the methodological quality. In addition, the Cochrane risk of bias tool was used to evaluate the quality of included studies, which contained 7 parts with a possible risk of bias, such as random sequence generation, allocation concealment, blinding of participants and personnel, blinding of outcome assessment, incomplete outcome data, selective reporting, and other bias. Each study was rated low, high risk of bias, or unclear (no reporting or missing information) according to experiment information in the article.

### Statistical Analysis

We used the Stata 16.0 (Stata Corp, College Station, TX, USA) and RevMan version 5.3 (Cochrane Collaboration, UK) software to perform all statistical analyses. The sample size and data of cognitive outcomes (mean change in cognitive scale scores from baseline to post-intervention) of the control and intervention groups were used to generate effective sizes (16), and standardized mean difference (SMD) and 95% confidence intervals (*CI*s) were calculated to eliminate the influence of different scale evaluation systems. For studies reporting multiple cognitive outcomes, effect sizes were calculated separately for each test and the overall effect size was obtained, which ensured that each study was represented by effect size (16). In addition, for the results with large scores indicating poor cognitive status, they were input into the software as negative numbers. The heterogeneity was assessed by the *Q*-test and *I*^2^ index. Besides, consistent with the guidelines in the Cochrane handbook, the value of *p* < 0.10 indicated statistically significant heterogeneity between studies. Then, the fixed-effects model and random-effects model were selected according to the heterogeneity of the results.

We first assessed whether PA exerted a significant beneficial effect on the overall cognitive function, and then sensitivity analysis was performed to test the robustness of the results. We conducted a subgroup analysis and a moderation analysis to explore the source of heterogeneity and possible factors that affected the results from the following aspects: whether the study included an overall cognitive assessment scale (Yes or No), time form to intervention (≤ 3 months or >3 months), whether there was cognitive impairment before intervention (Yes or No), type of measurement (subjective or objective), type of control (usual care without extra PA or intervention, such as PA components or intervention, including cognitive training components), type of intervention (aerobic exercise or strength/balance/stretching/physiotherapy or combined), intensity (high or moderate or low or mixed), duration of the intervention (<3 months or ≥3 months). Moderation effects were analyzed with the between-group heterogeneity (*Q*_B_) test, which provided an estimate of the between-groups variance (13). The value of *p* < 0.05 indicated statistically significant.

On this basis, we conducted a further analysis of the types of aerobic exercises to explore the effects of conventional formation and traditional exercises on cognitive function. At the same time, the change of specific domains of cognition before and after PA was explored, such as the executive ability (attention, processing speed, and set-shifting), working memory, spatial function, learning, and long-term memory. Moreover, we performed a series of meta-regression analyses to assess the effect of multiple regulatory variables on cognitive function, which involved age and the proportions of women, right hemiplegia and ischemic stroke, and the frequency of PA. Finally, the funnel plot and Egger's test were performed to evaluate the potential for publication bias for meta-analysis. Significance was set at *p* < 0.05 and *p* < 0.1 was reported as a trend.

## Results

According to the search strategy, the database searching produced 6,252 items and 2,067 duplicate records were removed. Therefore, the initial screening yielded 4,185 potentially relevant citations, 149 of them were retrieved for full-text review. Then, 127 studies were excluded for the following reasons: no cognitive outcome, inappropriate control group, and secondary analysis. Finally, 22 studies were included in quantitative synthesis, representing data from 1,601 participants (792 from the control group and 809 from the intervention group) (9–11, 14, 15, 17–33). Unlike the meta published in 2017, Nilsson's (34) study was not included because both the experimental group and control group received treadmill training, and the difference lies in whether there was weight support. Although the Functional Independence Measure (FIM) was used in Mead's study (35), there were no separate cognitive data, so the study was also excluded. Chen (36) trial was published in the Chinese language and cannot be searched in the included database. In addition, 11 latest trials that met the inclusion criteria were included for meta-analysis (10, 11, 14, 15, 17–19, 25, 27, 29, 33).

**Figure 1:**
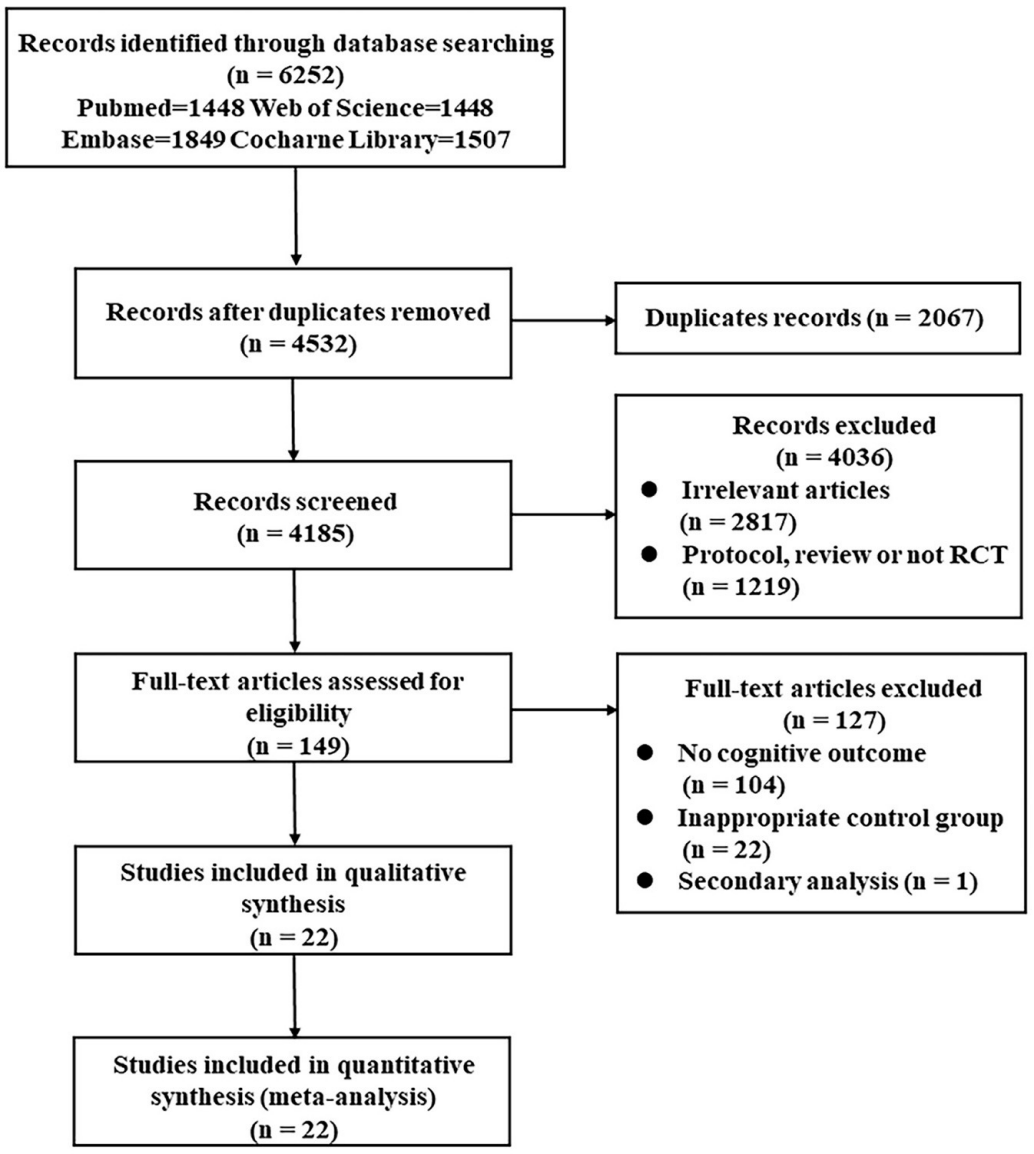
Preferred Reporting Items for Systematic Reviews and Meta-analysis (PRISMA) flow diagram of the search strategy. PRISMA, Preferred Reporting Items for Systematic Reviews and Meta-analysis; RCT, randomized controlled trial.

Besides, the flow diagram showed the detailed process of selection (Figure 1). In addition, the included articles were published between 2001 and 2019, and these studies came from a total of 13 countries, of which 4 studies were from China (17, 21, 25, 33), 4 were from Canada (10, 24, 27, 32), and 3 were from the United States (28, 29, 31). In terms of the participants' characteristics, the sample sizes of the intervention group ranged from 14 to 358, the average age was 62.86 years, and women comprised 40.26% of the sample. When it comes to cognitive function, 15 studies employed a global cognitive function assessment scale (9–11, 14, 15, 18–21, 23, 25–27, 31, 33), specifically the Barrow Neurological Institute screen (BNIS), the FIM, the Addenbrooke's Cognitive Examination Revised (ACE-R), the Mini-Mental Status Exam (MMSE), the ADAS-cog, the Montreal Cognitive Assessment (MOCA), the Loewenstein Occupational Therapy Cognitive Assessment (LOTCA), Raven's Progressive Matrices Test (RPMT), or the Stroke Impact Scale (SIS). For those scales that are not mainly aimed at detecting cognition, such as FIM and SIS, the data of global cognitive parts need to be listed separately. The data further showed that 12 of these studies adopted objective measures (9–11, 14, 15, 18, 20, 21, 25–27, 33), and 3 administered subjective cognitive assessments (19, 23, 31). Other 7 studies conducted multiple neuropsychological tests in different cognitive domains (17, 22, 24, 28–30, 32). In addition, the patients in the 14 studies showed cognitive impairment before the intervention (10, 14, 17, 20, 21, 23–27, 29–31, 33), while patients in other 8 articles did not show this.

Among the PA training conditions, the duration of intervention ranged from 4 to 72 weeks, the average duration lasted for 15 weeks and the average frequency was 3.8 times every week. Most notably, 10 trials consisted only of aerobic exercise training (10, 14, 18, 23, 25, 27, 28, 30, 32, 33), 2 of which adopted traditional exercises (25, 33). Moreover, 4 articles involved strength/balance/stretching/physiotherapy without a primary aerobic component (11, 21, 22, 26) and 8 studies included combined PA trainings (9, 15, 17, 19, 20, 24, 29, 31). From the perspective of the type of control group, 10 trials included control groups that received usual care, daily routines, or wait-list control, without any PA component (10, 11, 15, 17, 21–24, 31, 33). In addition, 9 studies contained control group that included PA components without a primary aerobic component (muscle relaxation, stretching, balance, and physiotherapy) (9, 14, 19, 20, 26–29, 32) and the rest of 3 studies recruited control groups that accepted additional intervention without any form of PA (rhythm-and-music-based therapy, cognitive training, and social communication) (18, 25, 30). Further details about the characteristics of included studies are shown in the Supplementary Table 1.

### Quality Assessment

The Cochrane bias tool was used to evaluate the methodological quality of each study. The results demonstrated that 3 (13.6%) studies were judged to have high risk in the blinding of participants and personnel, 1 (4.5%) study was regarded as high risk in the blinding of outcome assessment, and 1 (4.5%) study was considered as high risk in the sequence generation and the blinding of outcome assessment. Specifically, 2 (9.1%) studies did not report the method of sequence generation and 8 (36.4%) articles did not describe the means of allocation concealment. In the terms of the implementation of blinding, the blinding of participants and assessors was not reported in 15 (68.2%) and 7 (31.8%) trials, respectively. Finally, all (100.0%) studies have a low risk of incomplete outcome data and selective reporting bias and did not give information about other possible biases. Specific evaluation results were demonstrated in the Supplementary Table 2 and Supplementary Figure 1.

### Effects of PA on Global Cognitive Function

**Figure 2:**
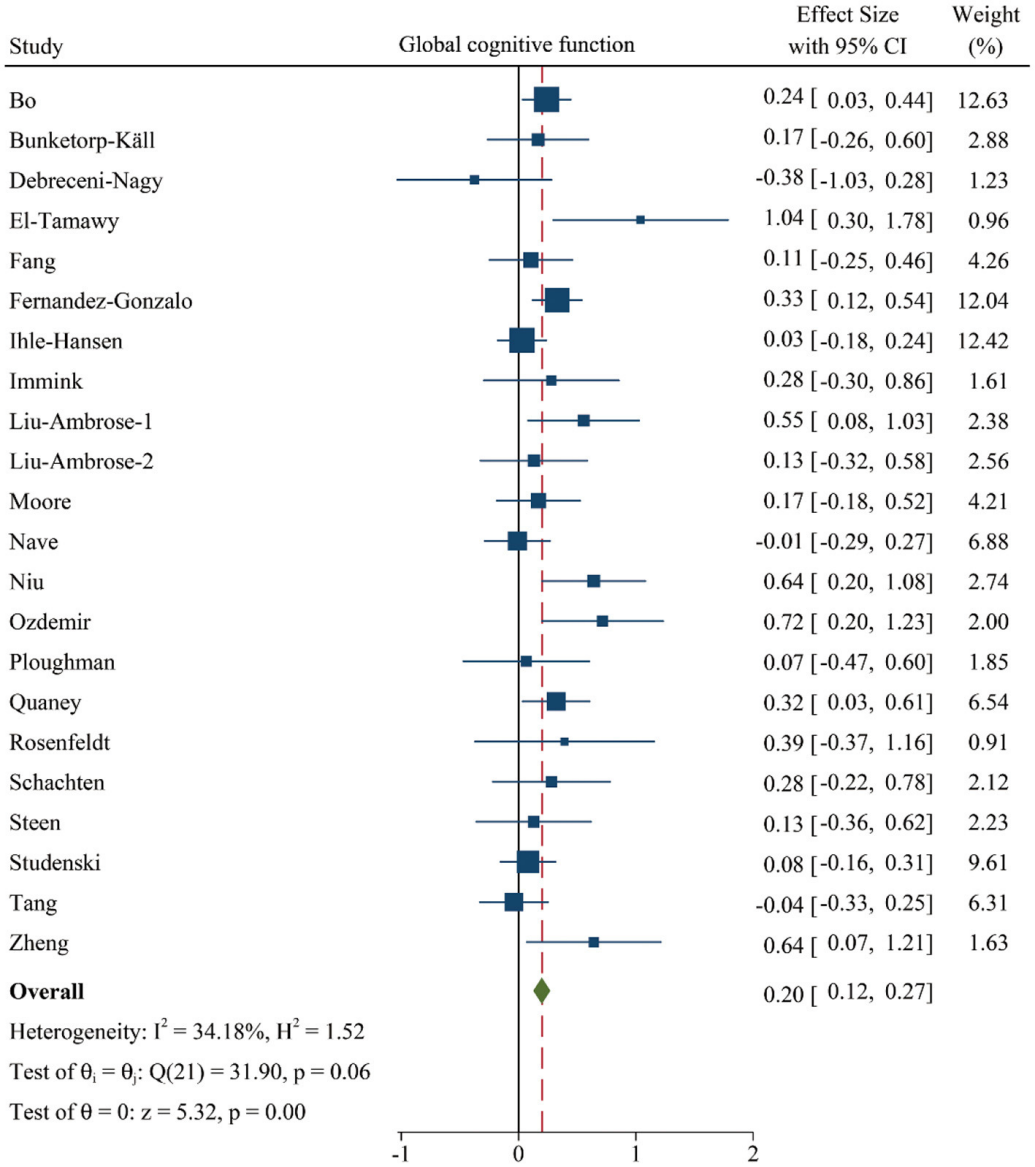
Forest plot of meta-analysis in the effect of PA on global cognitive function. Positive values of pooled effect size represented that PA improved cognitive performance. *CI*, confidence interval; PA, physical activity.

First, we investigated whether patients with cerebrovascular disorders can benefit from PA training, extracting data from 1,601 individuals in 22 studies. The fixed-effects model was performed and the results revealed that a significant and positive effect of PA training was observed in the performance of general cognition (SMD: 0.20 [95% *CI*: 0.12–0.27], *z* = 5.32, *p* < 0.001, Figure 2 and Supplementary Table 3). In addition, low-moderate heterogeneity between studies was observed in the Figure 2 and Supplementary Table 3.

### Sensitivity Analysis

Then, we conducted a sensitivity analysis and the figure showed that pooled effect size was not overaffected by the specific study, indicating the result was relatively robust (Supplementary Figure 2).

### Effects of Influencing Factors in the Efficacy of PA on Cognitive Function

Then, we performed a series of subgroup analyses to explore the potential sources of heterogeneity and moderation analysis to investigate relevant factors that affected the positive effect of PA training on cognitive function.

#### Global Cognitive Assessment Scale

**Figure 3:**
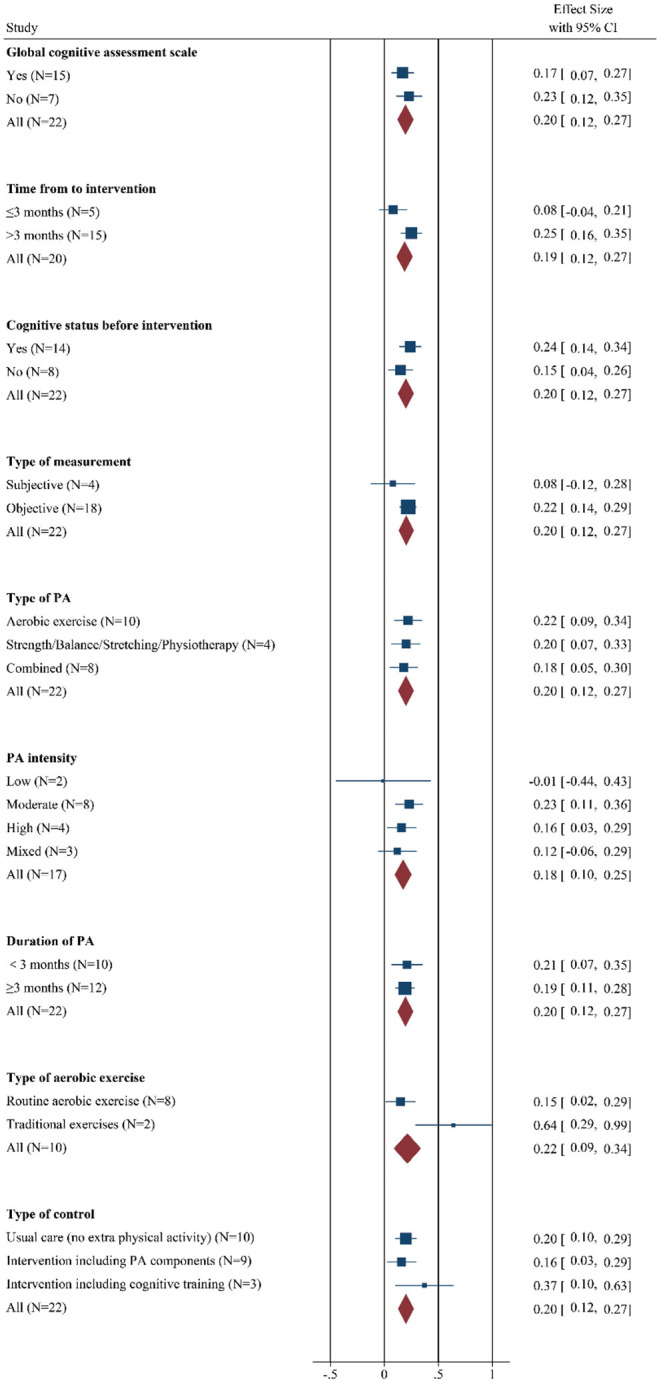
Forest plot of meta-analysis in the effect of regulatory factors on the global cognitive improvement by PA. Positive values of pooled effect size represented that PA improved cognitive performance. CI, confidence interval; PA, physical activity.

Since we included many studies involving multiple neuropsychological tests in different cognitive fields, we first discussed whether the study without overall cognitive function scales affected the meta-analysis results. The data suggested that the pooled effect size in the study with (SMD: 0.17 [95% *CI*: 0.07–0.27]) or without (SMD: 0.23 [95% *CI*: 0.12–0.35]) them all differed from zero, and there was no significance between these groups (*Q*_B_ = 0.76, *p* = 0.38, Figure 3 and Supplementary Figure 3), however, the study with general cognitive assessment showed moderate heterogeneity that may be regarded as a source of heterogeneity.

#### Time From Stroke to Intervention

In addition, the results indicated a significant and positive effect on the cognition of chronic cerebrovascular phase (SMD: 0.25 [95% *CI*: 0.16–0.35]), while the effect size of studied that received PA within 3 months was not different from zero (SMD: 0.08 [95% *CI*: −0.04–0.21]). However, effect size estimates were statistically homogenous within subgroups (*Q*_B_ = 4.60, *p* = 0.03, Figure 3 and Supplementary Figure 4).

#### Cognitive Status Before Intervention

Then, we explore the role of cognitive status before intervention, the figure demonstrated that compared with the patients without cognitive impairment (SMD: 0.15 [95% *CI*: 0.04–0.26]), the effect size was higher in the patients with cognitive deficits (SMD: 0.24 [95% *CI*: 0.14–0.34]). Moreover, significant heterogeneity was observed in the patients with worse baseline cognitive function, but it was not markedly higher than the other group. The interaction effect did not reach statistical significance (*Q*_B_ = 1.50, *p* = 0.22, Figure 3 and Supplementary Figure 5).

#### Type of Measurement

In the terms of the type of measurement, beneficial effects were observed among trials that adopted an objective cognitive scale (SMD: 0.22 [95% *CI*: 0.14–0.29]), while the pooled effect size for studies conducting subjective cognitive measures did not significantly differ from zero (SMD: 0.08 [95% *CI*: −0.12–0.28]). In addition, low-moderate heterogeneity was observed in the patients with objective cognitive assessment. There was no significance in the interaction effect between groups (*Q*_B_ = 1.47; *p* = 0.23, Figure 3 and Supplementary Figure 6).

#### Type of PA

Among intervention conditions, the choice of PA type has always been a controversial topic. In this meta-analysis, the results revealed that regardless of whether the patient received aerobic exercise (SMD: 0.22 [95% *CI*: 0.09–0.34]), strength/balance/stretching/physiotherapy (SMD: 0.20 [95% *CI*: 0.07–0.33]) or combined program (SMD: 0.18 [95% *CI*: 0.05–0.30]), their cognition gains similar extent improvement. Significant moderate heterogeneity was observed in the patients with strength/balance/stretching/physiotherapy, but it was not markedly higher than the other two groups, which may not be a strong source of heterogeneity. Besides, the interaction effect was not statistically significant (*Q*_B_ = 0.21, *p* = 0.90, Figure 3 and Supplementary Figure 7).

#### PA Intensity

In addition, different exercise intensities produced different beneficial effects on cognition. Specifically, participants introduced moderate-intensity PA experienced the largest cognitive benefits (SMD: 0.23 [95% *CI*: 0.11–0.36]), although cognitive gains were also apparent among subjects who underwent a high-intensity PA regimen (SMD: 0.16 [95% *CI*: 0.03–0.29]) and mixed intensity PA program (SMD: 0.12 [95% *CI*: −0.06–0.29]). The pooled effect size for trials that used low-intensity PA training was not significantly different from zero (SMD: −0.01 [95% CI: −0.44–0.43]). From the perspective of heterogeneity, there was abnormally moderate-high heterogeneity in trials adopting mixed intensity exercise, which may be regarded as the potentially relevant factors of heterogeneity. However, the between-groups difference did not result in statistical significance (*Q*_B_ = 2.06; *p* = 0.56, Figure 3 and Supplementary Figure 8).

#### Duration of PA

Clinically, PA training was usually considered as a chronic and long-term treatment option, however, this study reported that a longer intervention duration did not bring greater cognitive benefits relative to brief interventions (*Q*_B_ = 0.04; *p* = 0.83, Figure 3 and Supplementary Figure 9). In practical terms, PA duration within 3 months (SMD: 0.21 [95% *CI*: 0.07–0.35]) and interventions lasting at least (SMD: 0.19 [95% *CI*: 0.11–0.28]) were related with improvements in cognition, suggesting that there is no significant difference in their efficacy. Moreover, low-moderate heterogeneity was shown in the long-term PA groups, which was not different from the short-term training groups.

#### Type of Aerobic Exercise

Interestingly, in the 10 trials using aerobic exercise, there were 2 studies choosing traditional exercise and further subgroup analysis demonstrated a greater magnitude of cognitive gains in these groups (SMD: 0.64 [95% *CI*: 0.29–0.99]) than that in other routine aerobic exercise groups (SMD: 0.15 [95% *CI*: 0.02–0.29]). Besides, there was significance in the interaction effect between groups (*Q*_B_ = 6.52; *p* = 0.01, Figure 3 and Supplementary Figure 10).

#### Type of Control

Next, we study the effect of different control types on cognitive benefit after PA training. The control conditions were divided into 3 types: usual care (no extra PA), intervention including PA components, and intervention including cognitive training components. Unexpectedly, research with the control group involving cognitive components indicated a greater pool effect size (SMD: 0.37 [95% *CI*: 0.10–0.63]), while the trials containing control groups with usual care (SMD: 0.20 [95% *CI*: 0.10–0.29]) or with intervention, such as PA components (SMD: 0.16 [95% *CI*: 0.03–0.29]), showed little difference in the improvement of cognitive performance. Moderate heterogeneity was detected in trials that control groups involved PA components. However, the between-group difference was not statistically significant (*Q*_B_ = 1.90; *p* = 0.39, Figure 3 and Supplementary Figure 11).

#### Cognitive Domains

**Figure 4:**
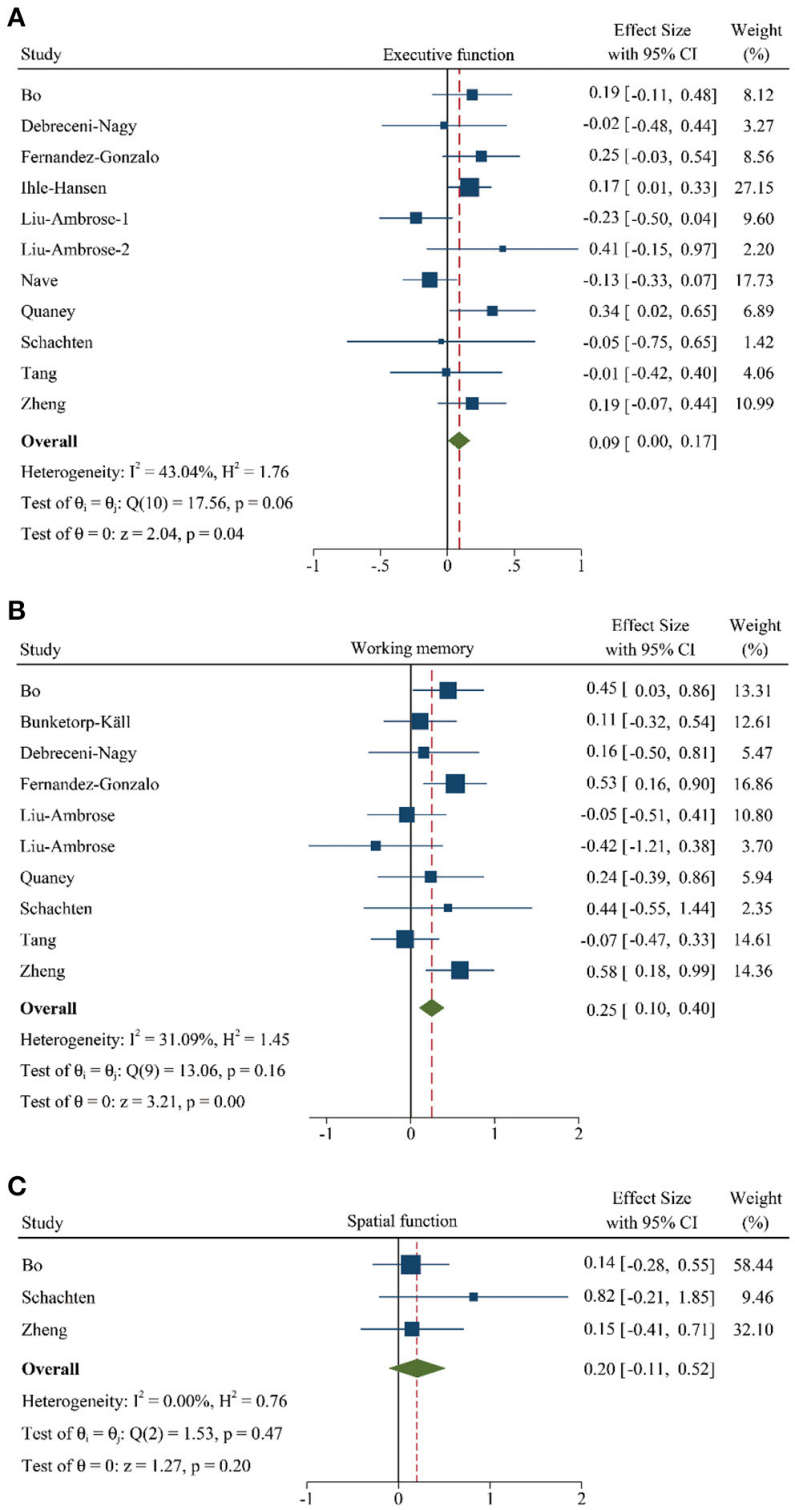
Forest plot of meta-analysis in the PA on specific cognitive domains. **(A)** Meta-analysis of effect of PA on executive function. **(B)** Meta-analysis of effect of PA on working memory. **(C)** Meta-analysis of effect of PA on spatial function. Positive values of pooled effect size represented that PA improved cognitive performance. **Abbreviation:** CI, confidence interval; PA, physical activity.

Meanwhile, to investigate the role of PA training on different cognitive domains, we divided the cognitive function into 3 subgroups according to the multiple neuropsychologist tests, such as executive function, working memory, and spatial function. We performed a meta-analysis from 11 studies (948 participants) (10, 11, 14, 17, 19, 22, 24, 28, 30, 32, 33), 10 trails (477 participants) (10, 17–19, 22, 24, 28, 30, 32, 33), and 3 different research (151 participants) (17, 30, 33), respectively. The results gave information about that the executive function (SMD: 0.09 [95% *CI*: 0.00–0.17], Figure 4) and working memory (SMD: 0.25 [95% *CI*: 0.10–0.40], Figure 4) were significantly improved after PA training. However, although spatial function showed a greater pooled effect size (SMD: 0.20 [95% *CI*: −0.11–0.52], Figure 4), indicating a positive effect, there was no statistical difference. The details of these results are summarized in Supplementary Table 3.

### Meta-Regression Analysis

**Figure 5:**
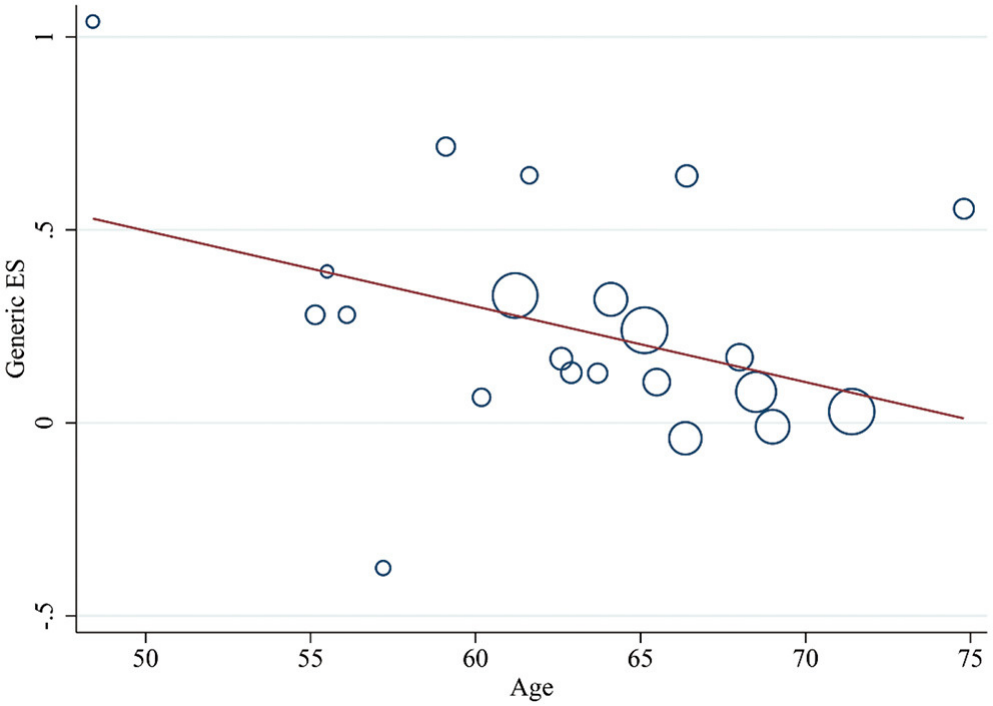
Meta-regression of age for the improvement of cognitive performance by PA. ES, effect size; PA, physical activity.

Meanwhile, a meta-regression analysis was performed to explore the regulatory factors of PA on the improvement of cognitive performance. This series of results found that gender, stroke type, hemiparetic side, and the frequency of intervention could not be regarded as effect factors to explain the heterogeneity between intervention and control groups (*p* > 0.05 for all results). In addition, it was worth noting that a negative relationship between age and cognition was reported (Coef: −0.02 [95% *CI*: −0.039–−0.0004], *P* = 0.046, Figure 5). The details were found in Supplementary Table 4.

### Publication Bias

**Figure 6:**
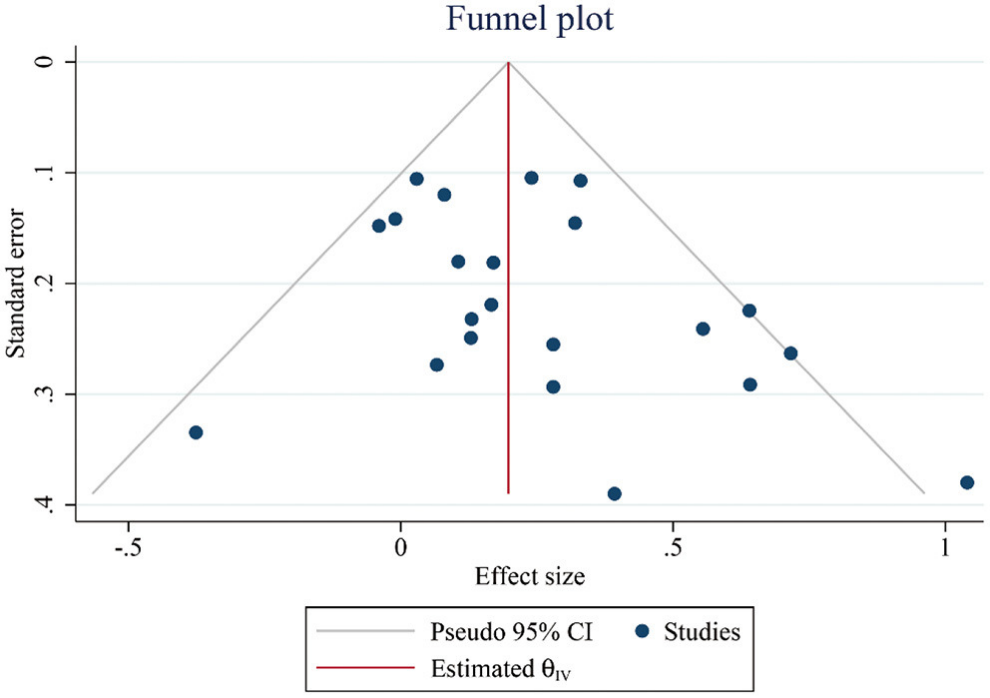
Funnel plots representing standard error (SE) and pooled effect size in the effect of PA on cognitive performance. CI, confidence interval; PA, physical activity.

Finally, we generated a funnel plot to make a preliminary judgment, and conducted Egger's test to confirm that there was no publication bias for the data of global cognitive function between intervention groups and control groups (*t* = 1.64, *p* > 0.05, Figure 6).

## Discussion

This study incorporating the latest data revealed that PA rescued the cognitive dysfunction of patients with cerebrovascular disorders, whether it was assessed by the global cognitive function scale or detected by neuropsychological tests in different cognitive fields. However, the use of objective assessments was more sensitive than the adoption of subjective scales in testing the PA-induced changes of cognitive performance, showing a larger pooled effect size. In addition, patients with baseline cognitive impairment achieved a greater degree of cognitive benefit from PA, compared with their cognitively normal control subjects. Even in the chronic phase of the disease (>3 months), a remarkable improvement of cognition was found in participants. In the terms of PA characteristics, different types and duration of PA did not show a significant correlation with the degree of cognitive performance. Interestingly, patients receiving traditional aerobic exercises seemed to get greater cognitive benefits than those with the routine formation of training. Moreover, PA with moderate intensity may be regarded as a better choice. Further regression analysis demonstrated that the beneficial effects of PA on cognition were negatively correlated with age. In addition, we clarified the impact of PA training on the specific domains of cognition, manifested as a significant and positive effect on executive function and working memory. Nevertheless, PA showed benefits for spatial function but was not statistically significant.

Cerebrovascular disorder is a type of disease with an extremely high disability and fatality rate. Previous studies have mostly focused on motor dysfunction, but increasing research has shown that VCI caused by cerebrovascular disease is a serious and progressively worsening cognitive impairment syndrome that deserves more attention. Clinically, there is currently no effective drug treatment for VCI. In addition, patients with VCI are often accompanied by psychiatric symptoms, and the abuse of antipsychotics may increase the risk of metabolic syndrome and death. Therefore, it is very essential to determine a widely accessible, valid, and cost-effective non-drug treatment.

In recent years, cumulative evidence has shown that PA was widely accepted as an indispensable alternative therapy in a variety of diseases. A cross-sectional study involving 1.2 million people reported that the various types of exercise were associated with lower mental health burdens. Among them, popular team sports, cycling, and aerobic and gym activities received greater benefits (37). Another prospective cohort study revealed that regular, moderate-to-high-intensity PA activities detected by object instruments can lead to a lower risk of atrial fibrillation and stroke (38). In addition, many studies have shown that PA exerted a positive effect on improving the cognitive impairment in the older adults and patients with Alzheimer's disease (AD) (39–42). However, in the research field of cerebrovascular disorders, the influence of PA training on cognitive dysfunction was still controversial. Although there was a meta-analysis suggesting that PA training shows a significant positive impact on cognition after stroke, in the past 5 years, 11 latest studies were published (10, 11, 14, 15, 17–19, 25, 27, 29, 33), including 3 studies with negative results (11, 14, 15). Therefore, we compiled the latest literature and updated the meta-analysis. The results from 22 studies found that PA significantly improved the general cognitive dysfunction, which was consistent with previous studies. Moreover, the study indicated that executive function and working memory received significant gains from PA training, which were the main impaired cognitive domain of VCI, and reported the improvement trend of PA on spatial function based on 3 studies (17, 30, 33). These findings supplemented the effect of PA training on cognitive performance in different fields, which has not been well reflected in previous studies. However, more randomized controlled studies adopting comprehensive and domain-specific cognitive assessment scales were needed to confirm these findings.

Further, we investigated the potential mediators that may affect the regulation of PA on general cognitive improvement through a series of moderation analyses. First of all, the results showed that pooled effect size remained stable regardless of whether the overall cognitive function measure or the domain-specific assessment scale was used. However, the adoption of objective detection resulted in a larger pooled effect size, which may be because the objective scale was more sensitive to PA-induced changes in cognitive function than the subjective test. In addition, patients with cognitive deficits derive more benefits from PA compared with counterparts with normal cognition. At the same time, most studies did not report the specific motor function of the subjects before the intervention but stated that the subjects could complete the PA required for their respective studies. Therefore, the interaction between the improvement of motor function and the improvement of cognitive function needs to be further investigated.

Clinically, early intervention after stroke can effectively improve the recovery of limb function in patients with ischemic stroke (43), and the potential mechanism was that PA training can upregulate the content of neurotrophic factors, reduce the volume of the lesion, and protect the surrounding tissues against the damage of oxidative stress and inflammation (44, 45). On the contrary, our research indicated that the cognitive function of patients who started PA training after 3 months has been restored to a greater extent, and the cognitive function of patients who receiving training within 3 months did not change significantly, which was consistent with the previous meta-analysis results. Generally speaking, the acute phase and recovery phase (within half a year) after a stroke were the most important periods for functional recovery. Most of the dysfunctions can be recovered well at this time. After 6 months, the recovery of various dysfunction will gradually slow down and reach a stable stage. However, this inconsistent result may be due to the improvement of motor function ensuring the benefits of PA training for cognitive function and these findings proved the advantage of PA in rescuing the cognitive function of patients with chronic cerebrovascular disease.

Among the intervention situation, different types of PA exerted the same degree of improvement in cognition. Previous studies have shown that the use of aerobic exercise, resistance training, or combined interventions can significantly improve the cognitive dysfunction of patients with stroke (10, 22, 46, 47). However, since the isolated effects of aerobic and resistance training were not compared with the combination of the two methods, it remained difficult to ensure that such combination induced a better beneficial effect on cognition after stroke. In addition, there was a study suggesting that the combination of aerobic and resistance exercise produced greater cognitive gains for the elderly compared with patients who only applied to aerobic exercise (48), and the combined intervention showed a better improvement in oxygen uptake at the ventilatory threshold, muscle strength, and lean mass in patients with stroke (49). At present, another view accepted by some people has emerged, which was that the combined intervention of PA and cognitive training achieved greater improvement on cognition compared with either training alone in patients with VCI (17). Therefore, more trials were needed to determine the selection priority of PA type for cognitive impairment induced by cerebrovascular diseases.

Then, in the terms of PA intensity, moderate levels showed a greater magnitude of amelioration in cognition, which was more suitable for patients with VCI and might be the most promising mode to alleviate VCI. High-intensity and mixed-intensity PA improved the cognitive ability, but low-intensity PA did not lead to significant improvement. However, due to the small number of studies, further research is needed to draw this conclusion. A meta-analysis of more than 44,000 middle-aged and elderly people revealed that 30–40 minutes of moderate to vigorous PA per day significantly reduced the association between sedentary time and the risk of all-cause mortality (50). Similarly, another study showed that PA improved the cognitive function in the over 50 years, regardless of the cognitive status of participants, and recommended that patients should engage in at least moderate-intensity PA (51). In previous studies, it was found that the long-term moderate-intensity exercise can effectively improve the cognitive function of patients with VCI (10, 33), and moderate-intensity swimming exercise can significantly improve the cognitive dysfunction in mice with chronic cerebral hypoperfusion (52). In this study, the pooled effect size of high-intensity PA on cognition was lower than that of moderate-intensity PA, which may be due to the limitation of the study numbers and the influence of other exercise parameters. Interestingly, several trials investigated the influence of a single exercise and reported that a single moderate-intensity aerobic exercise increased brain-derived neurotrophic factor (BDNF) levels (53), and improved cognitive control and attention (54). On the contrary, a study proposed that high-intensity interval exercise can significantly enhance the gait ability of patients with chronic stroke and improve cardiovascular function (55). Therefore, further research was needed on the effects and mechanisms of the different intensities of PA on cognition in patients with cerebrovascular disease.

Notably, in the aerobic exercise subgroups, 2 trials involving traditional exercise were extracted separately for statistical analysis (25, 33). The results indicated that stroke survivors who received traditional exercise exerted greater cognitive benefits, but at present, the interpretation of the results needs to be cautious with a limited number of studies. The meta-analysis found that longer intervention duration did not bring better efficacy but showed a treatment effect consistent with the intervention time within 3 months. Further data explained that in the included studies, there was no significant correlation between the effect size and the frequency of intervention. Nevertheless, we found that in the trials with intervention time longer than 3 months, the weekly intervention frequency was 3.4 times, which was significantly <4.2 times in the study with duration <3 months, proving that the mutual restriction between frequency and duration limited the drawing of more in-depth conclusions.

For a long time, the type, intensity, duration, and frequency of exercise are the most critical elements in the formulation of exercise programs. The key to making an excellent exercise program is to formulate personalized treatment plans based on the specific functional assessment of different subjects, rather than simply and mechanically adjusting these parameters. A study suggested that increased adherence to the intervention was associated with improved cognitive function (11). Another trial independent of these parameters showed that moderate-intensity exercise can upregulate serum BDNF and improve working memory or executive function in older adults. However, this beneficial effect is affected by intermittent light walking exercises in sitting after exercise (56).

We further explored the long-term effects of PA and found that 9 studies took 1–7 months of follow-up to observe the long-term effects of PA (10, 14, 17, 18, 21, 27–29, 33). The results of 6 studies supported that the benefits of PA on cognitive performance were sustained (14, 21, 27–29, 33), but some studies showed it on overall cognitive function, several studies showed it on different cognitive subdomains, and another 3 studies did not show the long-term effects of PA (10, 17, 18). Therefore, the current research has not been able to draw relatively consistent conclusions about the time dependence of PA, which is also an important future research direction to determine the temporal changes in the beneficial effects of PA on cognition.

Finally, we explored the influence of the control type on the results. Participants receiving PA with cognitive training obtained a larger pooled effect size, while studies that included unusual care and intervention with exercise components showed a similar degree of improvement that may be a consequence of the small number of studies.

In the regression analysis, although the results concluded that gender, stroke type, and hemiplegic side did not excessively affect the results, the effectiveness of the PA training on cognitive function was negatively correlated with age. Studies have shown that the prevalence of vascular dementia increased with age (57). Similarly, stroke mortality induced by low PA increased with age, and the risk was higher in men than women (58). In addition, a study reported that among post-stroke survivors with high mean age, PA did not show significant improvements in cognition (11). The above results suggested that age was a pivotal risk factor for cerebrovascular diseases and cognitive ability achieved less benefit in older adult patients, therefore, early prevention was considered as a better choice.

## Limitation

Methodological limitations were observed in this meta-analysis to a certain extent with 6 studies having a high risk of bias in some aspects, and in the extraction and collection of subject characteristics, some articles did not report the relevant information, such as educational background, compliance, motor function, and baseline fitness level. Therefore, the impact of these potential confounding factors for PA on cognitive performance cannot be investigated based on current studies, but may be worth paying attention to. In addition, in the moderation analysis, several results only contained a small number of studies, and the fewer sample size limited the precision of pooled estimates and the stability of the results. More trials with a larger sample size were needed to increase the credibility and reliability of the results.

## Summary

In conclusion, this updated review confirmed that PA training can significantly improve overall cognitive ability, but the positive effect on cognition was offset with the increasing age. PA was effective in the prevention and treatment of cognitive impairment in patients with cerebrovascular diseases, even in the chronic stage. Moderate-intensity PA was considered as a more potential choice. This study also clarified the influence of PA training on specific cognitive domains, manifested as a positive impact on executive function and working memory. These findings contributed to the development of personalized intervention schemes to maximize the recovery of cognitive function and strengthened the evidence that PA held promise as an effective non-drug therapy for VCI.

## Data Availability Statement

The original contributions presented in the study are included in the article/Supplementary Material, further inquiries can be directed to the corresponding author/s.

## Author Contributions

HLin, HLiu, WL, and LC contributed to the study design. HLin, HLiu, YD, and XY were responsible for study searching, processing of the data, and drawing figures and tables. HLin and HLiu contributed to the analysis and interpretation of the data. HLin, HLiu, and WL contributed to writing manuscripts. ZL, LY, JT, WL, and LC were in charge of revising the article. All authors contributed to the article and approved the submitted version.

## Funding

This study was supported by grants from the Key Project of National Natural Science Foundation of China (Grant No. 82030123) and the Youth top talent project of Fujian Province—Young Eagle Project (No. 2901-750102003).

## Conflict of Interest

The authors declare that the research was conducted in the absence of any commercial or financial relationships that could be construed as a potential conflict of interest.

## Publisher's Note

All claims expressed in this article are solely those of the authors and do not necessarily represent those of their affiliated organizations, or those of the publisher, the editors and the reviewers. Any product that may be evaluated in this article, or claim that may be made by its manufacturer, is not guaranteed or endorsed by the publisher.
