# Supplementary material for: Effect of Physical Activity on Cognitive Impairment in Patients With Cerebrovascular Diseases: A Systematic Review and Meta-Analysis
Source: Front Neurol. 2022 May 6;13:854158. doi: 10.3389/fneur.2022.854158 (PMC9120585; doi:10.3389/fneur.2022.854158)
Supplement: Supplementary file 1 [file Table_1.docx]

**Supplementary Table 1 Characteristics of included studies in the systematic review and meta-analysis.**

| **Study** | **Year** | **Country** | **Group** | **N** | **Age (years)** | **Sex (F, %)** | **Time from to intervention** | **Cognitive outcomes** | **Type of PA** | **PA session duration** | **Duration of PA** | **Frequency of PA** |
| --- | --- | --- | --- | --- | --- | --- | --- | --- | --- | --- | --- | --- |
| Bo | 2019 | China | Con | 47 | 64.36 (2.31) | 20 (42.55) | Medically stable, less than six months post-stroke. | TMT-B, Stroop color-word, Forward digit span, MRT | Combined | 50min/d | 12 weeks | 3x/week |
|  |  |  | PA | 42 | 65.12 (2.56) | 19 (45.24) |  |  |  |  |  |  |
| Bunketorp-Käll | 2017 | Sweden | Con | 41 | 63.7 (6.7) | 19 (46.3) | Days: 1096.3 (439) | BNIS | Horse-riding therapy | 240 min/session | 12 weeks | 2x/week |
|  |  |  | PA | 41 | 62.6 (6.5) | 17 (41.5) | Days: 1101.9 (576) |  |  |  |  |  |
| Debreceni-Nagy | 2019 | Hungary | Con | 16 | Median (IQR), 62 (52.75–68.25) | 5 (31.3) | Months: median (IQR), 13 (3–26.5) | FIM-cognitive subtest | Combined | 30 min/d | 4 weeks | 5x/week |
|  |  |  | PA | 19 | Median (IQR), 59 (50–63) | 6 (31.6) | Months: median (IQR), 10 (4.5–13.5) |  |  |  |  |  |
| El-Tamawy | 2014 | Egypt | Con | 15 | 49.67 ± 6.98 | 5 (33.3) | 3–18 months | ACE-R | Combined | 75 min/day | 8 weeks | 3x/week |
|  |  |  | PA | 15 | 48.4 ± 6.39 | 4 (26.7) |  |  |  |  |  |  |
| Fang | 2003 | China | Con | 78 | 61.8 ± 10.94 | 34 (43.6) | Less than one week | MMSE | Physiotherapy | 45 min/day | 4 weeks | 5x/week |
|  |  |  | PA | 50 | 65.49 ± 10.94 | 17 (34) |  |  |  |  |  |  |
| Fernandez-Gonzalo | 2016 | Spain | Con | 15 | 65.7 (12.7) | 4 (26.7) | Year: 4.3 (4.9) | Digits Span, RAVLT, CPT, Spatial Span, Stroop Word and Color, TMT-A and -B, FAS | Resistance Training | 4 sets of 7 maximal repetitions | 12 weeks | 2x/week |
|  |  |  | PA | 14 | 61.2 (9.8) | 3 (21.4) | Year: 3.5 (3.6) |  |  |  |  |  |
| Ihle-Hansen | 2019 | Norwegian | Con | 185 | 72.0 (11.3) | 65 (35.1) | Acute and subacute phase | MMSE | physical activity | physical activity: 30min/day  vigorous physical exercise: 45-60min/day | 72 weeks | physical activity: 5x/week  vigorous physical exercise: 2-3x/week |
|  |  |  | PA | 177 | 71.4 (11.3) | 78 (44.1) |  |  |  |  |  |  |
| Immink | 2014 | Australia | Con | 11 | 63.2 (17.4) | 8 (72.7) | Months: 23.3 (12.5) | SIS (memory and communication domain) | Yoga | group classes: 90 min/day individual home practice: 40 min/day | 10 weeks | group classes: 1x/week individual home practice: 5x/week |
|  |  |  | PA | 11 | 56.1 (13.6) | 5 (45.5) | Months: 81.6 (77.5) |  |  |  |  |  |
| Liu-Ambrose-1 | 2016 | Canada | Con | 35 | 73.7 (8.3) | 17 (49) | - | ADAS-cog | Aerobic training | 60 min/day | 24 weeks | 5x/week |
|  |  |  | PA | 35 | 74.8 (8.4) | 19 (54) |  |  |  |  |  |  |
| Liu-Ambrose-2 | 2015 | Canada | Con | 14 | 66.9 (9.0) | 3 (21.4) | Years: 2.9 (1.1) | Stroop color-words condition -Stroop C, Trail B – Trail A  Digit Forward – Digit Backward | Combined | 60 min/day | 24 weeks | 2x/week |
|  |  |  | PA | 11 | 62.9 (12.1) | 7 (63.6) | Years: 2.4 (1.0) |  |  |  |  |  |
| Moore | 2015 | UK | Con | 20 | 70 ± 11 | 4 (20) | Months:16 ± 12 | ACE-R, SIS (memory and communication domain) | Combined | 45-60 min/day | 19 weeks | 3x/week |
|  |  |  | PA | 20 | 68 ± 8 | 2 (10) | Months:21 ± 34 |  |  |  |  |  |
| Nave | 2019 | Germany | Con | 95 | 70 (11) | 36 (38) | Days: median (IQR), 27 (17-41) | MoCA | aerobic physical fitness training | 50 min/session | 4 weeks | 5x/week |
|  |  |  | PA | 105 | 69 (12) | 45 (43) | Days: median (IQR), 30 (17-39) |  |  |  |  |  |
| Niu | 2019 | China | Con | 20 | 66.4 ± 4.5 | 10 (50) | Lasted for 3 months or more | MoCA, LOTCA | Breath Qigong | 60 min/day | 12 weeks | 5x/week |
|  |  |  | PA | 20 | 66.4 ± 3.6 | 7 (35) |  |  |  |  |  |  |
| Ozdemir | 2001 | Turkey | Con | 30 | 61.8±9.2 | 11 (36.7) | Days: 36 | MMSE | Stretching, toning, neuromuscular facilitation exercises | 120 min/day | 9 weeks | 5x/week |
|  |  |  | PA | 30 | 59.1±5.9 | 9 (30) | Days: 41 |  |  |  |  |  |
| Ploughman | 2019 | Canada | Con | 27 | 66.5 (9.0) | 7 (25.9) | Months: 43.5 (36.1) | RPMT | Treadmill | 50-70 min/session | 10 weeks | 3x/week |
|  |  |  | PA | 25 | 60.2 (12.8) | 9 (36) | Months: 38.4 (44.2) |  |  |  |  |  |
| Quaney | 2009 | USA | Con | 19 | 58.96 (14.68) | 12 (63.2) | Years: 5.11 ± 3.53 | WCST, Stroop task, TMT B-A, SRTT | Aerobic Exercise | 45 min/session | 8 weeks | 3x/week |
|  |  |  | PA | 19 | 64.10 (12.30) | 9 (47.4) | Years: 4.62 ± 3.21 years |  |  |  |  |  |
| Rosenfeldt | 2019 | USA | Con | 8 | 58±12 | 1 (12.5) | Months: 21.9±20.6 | SIS domains-cognitive composite | Combined | 90 min/session | 4 weeks | 6x/week |
|  |  |  | PA | 32 | 55.5±13.6 | 10 (31.3) | Months: 15.8±13.6 |  |  |  |  |  |
| Schachten | 2015 | Germany | Con | 7 | 53.14 (13.54) | - | Years: 3.86 (1.67) | Go/No-Go task, BTT, MRT | Golf training | 60 min/session | 10 weeks | 2x/week |
|  |  |  | PA | 7 | 55.14 (17.64) |  | Years: 3.43 (1.81) |  |  |  |  |  |
| Steen | 2019 | Denmark | Con | 32 | 63.7 ± 9.2 | 6 (18.9) | - | MoCA | Combined | 15 min/day | 12 weeks | 5x/week |
|  |  |  | PA | 31 | 63.7 ± 8.9 | 8 (25.8) |  |  |  |  |  |  |
| Studenski | 2005 | USA | Con | 49 | 70.4 (11.3) | 22 (44.9) | Days: 74.1 (27.2) | FIM-Cognitive score, SIS (memory and communication domain) | Combined | 90 min/session | 12 weeks | 3x/week |
|  |  |  | PA | 44 | 68.5 (9.0) | 21 (47.7) | Days: 77.5 (28.7) |  |  |  |  |  |
| Tang | 2016 | Canada | Con | 25 | median (IQR): 64 (62–75) | 10 (40) | Years: median (IQR), 2.3 (1.8–5.1) | Verbal Digit Span Test, TMT B, Color-Word Stroop Test | Aerobic Exercise | 60 min/day | 24 weeks | 3x/week |
|  |  |  | PA | 25 | median (IQR): 66 (62–71) | 11 (44) | Years: median (IQR), 3.5 (2.2–6.7) |  |  |  |  |  |
| Zheng | 2020 | China | Con | 24 | 62.75±6.41 | 2 (8.3) | Months: 6.67±2.26 | MoCA | Baduanjin | 40 min/day | 24 weeks | 3x/week |
|  |  |  | PA | 24 | 61.63±9.21 | 5 (20.8) | Months: 6.50±2.06 |  |  |  |  |  |

**Abbreviation:** ACE-R, Addenbrooke’s Cognitive Examination- Revised; ADAS-cog, Alzheimer’s Disease Assessment Scale-Cognitive section; BNIS, Barrow Neurological Institute screen; BTT, Block-Tapping task; Con, control; CPT, Conners Continuous Performance; FAS, Verbal Fluency Test; FIM, Functional Independence Measure; IQR, inter quartile range; LOTCA, Loewenstein Occupational Therapy Cognitive Assessment; MMSE, Mini-mental state examination; MoCA, Montreal Cognitive Assessment; MRT, Mental Rotation Test; PA, physical activity; RAVLT, Rey Auditory Verbal Learning Test; RMPT, Raven’s Progressive Matrices Test; SIS, stroke impact scale; SRTT, Serial Reaction Timed Task; TMT, Trail Making Test; WCST, Wisconsin Card Sorting Task.
