# Supplementary material for: Effect of Physical Activity on Cognitive Impairment in Patients With Cerebrovascular Diseases: A Systematic Review and Meta-Analysis
Source: Front Neurol. 2022 May 6;13:854158. doi: 10.3389/fneur.2022.854158 (PMC9120585; doi:10.3389/fneur.2022.854158)
Supplement: Supplementary file 2 [file Table_2.docx]

**Supplementary Table 2 Results of quality evaluation of included studies according to the Cochrane bias tool.**

| Study | Sequence generation | Allocation concealment | Blinding of participants and personnel | Blinding of outcome assessment | Incomplete outcome data | Selective reporting | Other bias |
| --- | --- | --- | --- | --- | --- | --- | --- |
| Bo et al. | Low | Low | High | Low | Low | Low | NR |
| Bunketorp-Käll et al. | Low | Low | Low | High | Low | Low | NR |
| Debreceni-Nagy et al. | Low | NR | High | NR | Low | Low | NR |
| El-Tamawy et al. | NR | NR | NR | NR | Low | Low | NR |
| Fang et al. | Low | Low | NR | Low | Low | Low | NR |
| Fernandez-Gonzalo et al. | Low | Low | NR | NR | Low | Low | NR |
| Ihle-Hansen et al. | Low | Low | NR | NR | Low | Low | NR |
| Immink et al. | Low | Low | NR | Low | Low | Low | NR |
| Liu-Ambrose-1 et al. | Low | Low | Low | Low | Low | Low | NR |
| Liu-Ambrose-2 et al. | Low | Low | Low | Low | Low | Low | NR |
| Moore et al. | Low | NR | Low | Low | Low | Low | NR |
| Nave et al. | Low | NR | NR | Low | Low | Low | NR |
| Niu et al. | Low | Low | NR | NR | Low | Low | NR |
| Ozdemir et al. | High | NR | NR | High | Low | Low | NR |
| Ploughman et al. | Low | Low | NR | Low | Low | Low | NR |
| Quaney et al. | Low | NR | NR | Low | Low | Low | NR |
| Rosenfeldt et al. | Low | Low | NR | NR | Low | Low | NR |
| Schachten et al. | Low | NR | NR | NR | Low | Low | NR |
| Steen et al. | Low | Low | NR | Low | Low | Low | NR |
| Studenski et al. | NR | NR | High | Low | Low | Low | NR |
| Tang et al. | Low | Low | NR | Low | Low | Low | NR |
| Zheng et al. | Low | Low | NR | Low | Low | Low | NR |

**Abbreviation:** NR, not reported.
