# Supplementary material for: Effect of Physical Activity on Cognitive Impairment in Patients With Cerebrovascular Diseases: A Systematic Review and Meta-Analysis
Source: Front Neurol. 2022 May 6;13:854158. doi: 10.3389/fneur.2022.854158 (PMC9120585; doi:10.3389/fneur.2022.854158)
Supplement: Supplementary file 3 [file Table_3.docx]

**Supplementary Table 3 Results of all meta-analysis.**

| **Model** | **N** | **SMD** | **95%CI** | **P-value** | **Heterogeneity**  **(Q)** | **Q**  **P-value** | **I^2^** | **Q_B_** | **Q_B_**  **P-value** |
| --- | --- | --- | --- | --- | --- | --- | --- | --- | --- |
| **Overall** | **22** | **0.20** | **(0.12, 0.27)** | **<0.001** | **31.90** | **0.06** | **34.18** |  |  |
| **Global cognitive assessment scale** | **22** | **0.20** | **(0.12, 0.27)** |  | **31.90** | **0.06** | **34.18** | **0.76** | **0.38** |
| Yes | 15 | 0.17 | (0.07, 0.27) | <0.001 | 26.17 | 0.02 | 46.49 |  |  |
| No | 7 | 0.23 | (0.12, 0.35) | <0.001 | 4.98 | 0.55 | 0 |  |  |
| **Time from stroke to intervention** | **20** | **0.19** | **(0.12, 0.27)** |  | **29.60** | **0.06** | **35.80** | **4.60** | **0.03** |
| ≤3 months | 5 | 0.08 | (-0.04, 0.21) | 0.182 | 6.50 | 0.17 | 38.42 |  |  |
| >3 months | 15 | 0.25 | (0.16, 0.35) | <0.001 | 18.51 | 0.18 | 24.35 |  |  |
| **Cognitive status before intervention** | **22** | **0.20** | **(0.12, 0.27)** |  | **31.90** | **0.06** | **34.18** | **1.50** | **0.22** |
| Yes | 14 | 0.24 | (0.14, 0.34) | <0.001 | 20.76 | 0.08 | 37.38 |  |  |
| No | 8 | 0.15 | (0.04, 0.26) | 0.005 | 9.65 | 0.21 | 27.43 |  |  |
| **Type of measurement** | **22** | **0.20** | **(0.12, 0.27)** |  | **31.90** | **0.06** | **34.18** | **1.47** | **0.23** |
| Subjective | 4 | 0.08 | (-0.12, 0.28) | 0.414 | 2.96 | 0.40 | 0 |  |  |
| Objective | 18 | 0.22 | (0.14, 0.29) | <0.001 | 27.47 | **0.05** | 38.12 |  |  |
| **Type of PA** | **22** | **0.20** | **(0.12, 0.27)** |  | **31.90** | **0.06** | **34.18** | **0.21** | **0.90** |
| Aerobic exercise | 10 | 0.22 | (0.09, 0.34) | <0.001 | 14.18 | 0.12 | 36.55 |  |  |
| Strength/Balance/  Stretching/Physiotherapy | 4 | 0.20 | (0.07, 0.33) | 0.003 | 8.21 | 0.04 | 63.47 |  |  |
| Combined | 8 | 0.18 | (0.05, 0.30) | 0.006 | 9.30 | 0.23 | 24.72 |  |  |
| **PA intensity** | **17** | **0.18** | **(0.10, 0.25)** |  | **23.40** | **0.10** | **31.64** | **2.06** | **0.56** |
| Low | 2 | -0.01 | (-0.44, 0.43) | 0.98 | 2.17 | 0.14 | 54.01 |  |  |
| Moderate | 8 | 0.23 | (0.11, 0.36) | <0.001 | 7.68 | 0.36 | 8.86 |  |  |
| High | 4 | 0.16 | (0.03, 0.29) | 0.019 | 4.80 | 0.19 | 37.56 |  |  |
| Mixed | 3 | 0.12 | (-0.06, 0.29) | 0.183 | 6.68 | 0.04 | 70.07 |  |  |
| **Duration of PA** | **22** | **0.20** | **(0.12, 0.27)** |  | **31.90** | **0.06** | **34.18** | **0.04** | **0.83** |
| ＜3 months | 10 | 0.21 | (0.07, 0.35) | 0.003 | 15.49 | 0.08 | 41.89 |  |  |
| ≥3 months | 12 | 0.19 | (0.11, 0.28) | <0.001 | 16.37 | 0.13 | 32.81 |  |  |
| **Type of aerobic exercise** | **10** | **0.22** | **(0.09, 0.34)** |  | **14.18** | **0.12** | **36.55** | **6.52** | **0.01** |
| Routine aerobic exercise | 8 | 0.15 | (0.02, 0.29) | 0.02 | 7.66 | 0.36 | 8.63 |  |  |
| Traditional exercises | 2 | 0.64 | (0.29, 0.99) | <0.001 | 0 | 1 | 0 |  |  |
| **Type of control** | **22** | **0.20** | **(0.12, 0.27)** |  | **31.90** | **0.06** | **34.18** | **1.90** | **0.39** |
| Usual care (no extra physical activity) | 10 | 0.20 | (0.10, 0.29) | <0.001 | 10.22 | 0.33 | 11.91 |  |  |
| Intervention including PA components | 9 | 0.16 | (0.03, 0.29) | 0.017 | 17.35 | 0.03 | 53.90 |  |  |
| Intervention including cognitive training | 3 | 0.37 | (0.10, 0.63) | 0.006 | 2.43 | 0.30 | 17.83 |  |  |
| **Cognitive Domains** |  |  |  |  |  |  |  |  |  |
| Executive function | 11 | 0.09 | (0.00, 0.17) | 0.04 | 17.56 | 0.06 | 43.04 |  |  |
| Working memory | 10 | 0.25 | (0.10, 0.40) | <0.001 | 13.06 | 0.16 | 31.09 |  |  |
| Spatial function | 3 | 0.20 | (-0.11, 0.52) | 0.20 | 1.53 | 0.47 | 0 |  |  |

**Abbreviation:** CI, confidence interval; PA, physical activity.
