# Supplementary material for: Effect of Physical Activity on Cognitive Impairment in Patients With Cerebrovascular Diseases: A Systematic Review and Meta-Analysis
Source: Front Neurol. 2022 May 6;13:854158. doi: 10.3389/fneur.2022.854158 (PMC9120585; doi:10.3389/fneur.2022.854158)
Supplement: Supplementary file 4 [file Table_4.docx]

**Supplementary Table 4 Results of meta-regression analysis.**

| **Moderators** | **N** | **Coef.** | **95%CI** | **P-value** |
| --- | --- | --- | --- | --- |
| Sex (F, %) | 21 | -0.003 | (-0.012, 0.005) | 0.410 |
| Age | 22 | -0.020 | (-0.039, -0.0004) | 0.046 |
| Stroke type (ischemia, %) | 14 | -0.002 | (-0.008, 0.004) | 0.511 |
| Hemiparetic side (right, %) | 12 | -0.007 | (-0.019, 0.004) | 0.197 |

**Abbreviation:** CI, confidence interval; coef. correlation coefficient; PA, physical activity.
