# Supplementary material for: Effect of Physical Activity on Cognitive Impairment in Patients With Cerebrovascular Diseases: A Systematic Review and Meta-Analysis
Source: Front Neurol. 2022 May 6;13:854158. doi: 10.3389/fneur.2022.854158 (PMC9120585; doi:10.3389/fneur.2022.854158)
Supplement: Supplementary file 5 [file Data_Sheet_1.docx]

**Supplementary Figure 1 Quality evaluation of included studies according to the Cochrane bias tool.**

**

**

**Supplementary Figure 2 Sensitivity analysis in the effect of PA on cognitive performance.**


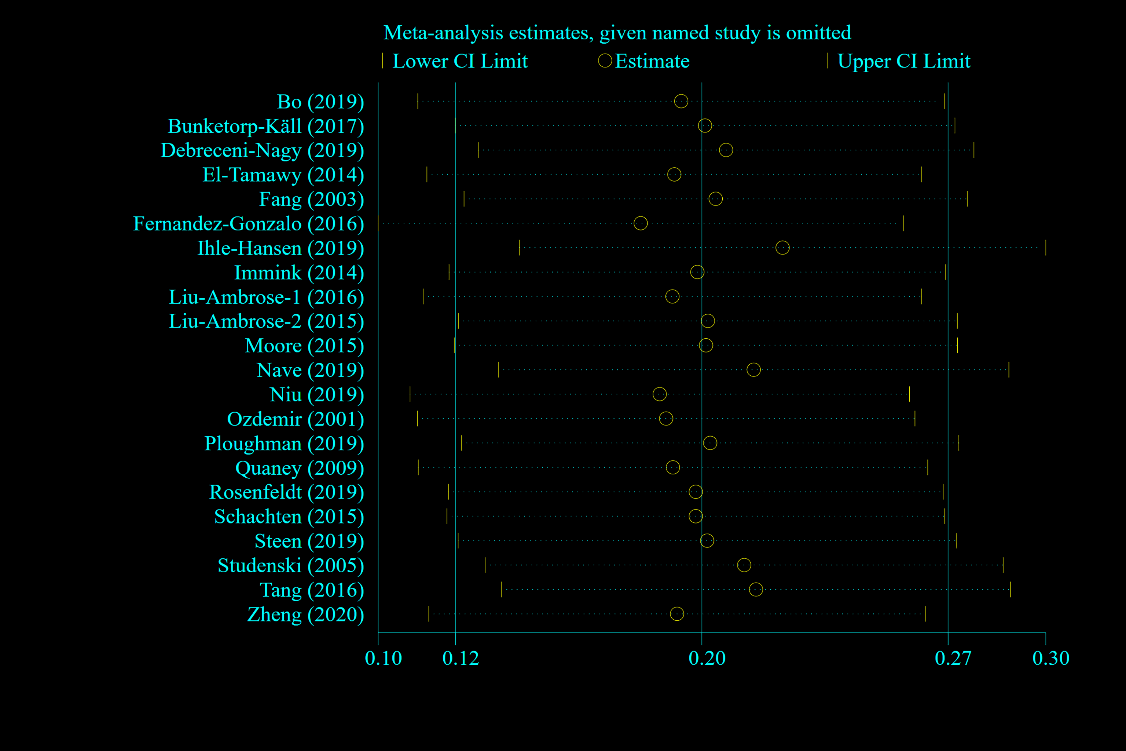
Abbreviation: CI, confidence interval; PA, physical activity.

**Supplementary Figure 3 Forest plot in the effect of global cognitive assessment scale on the cognitive gains by PA.** Positive values of pooled effect size represented that PA improved cognitive performance.


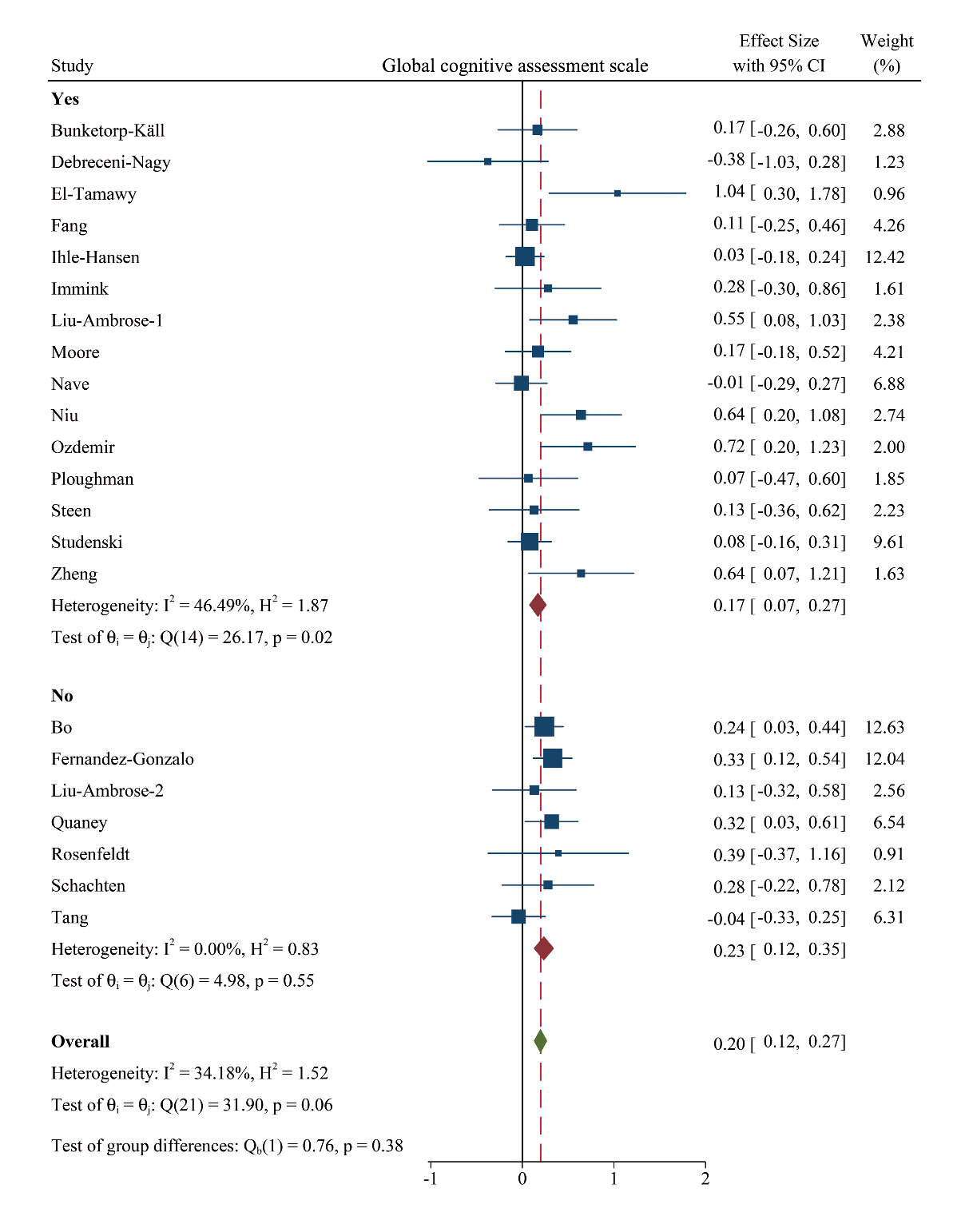
Abbreviation: CI, confidence interval; PA, physical activity.

**Supplementary Figure 4 Forest plot in the effect of duration of disease before intervention on the cognitive gains by PA.** Positive values of pooled effect size represented that PA improved cognitive performance.


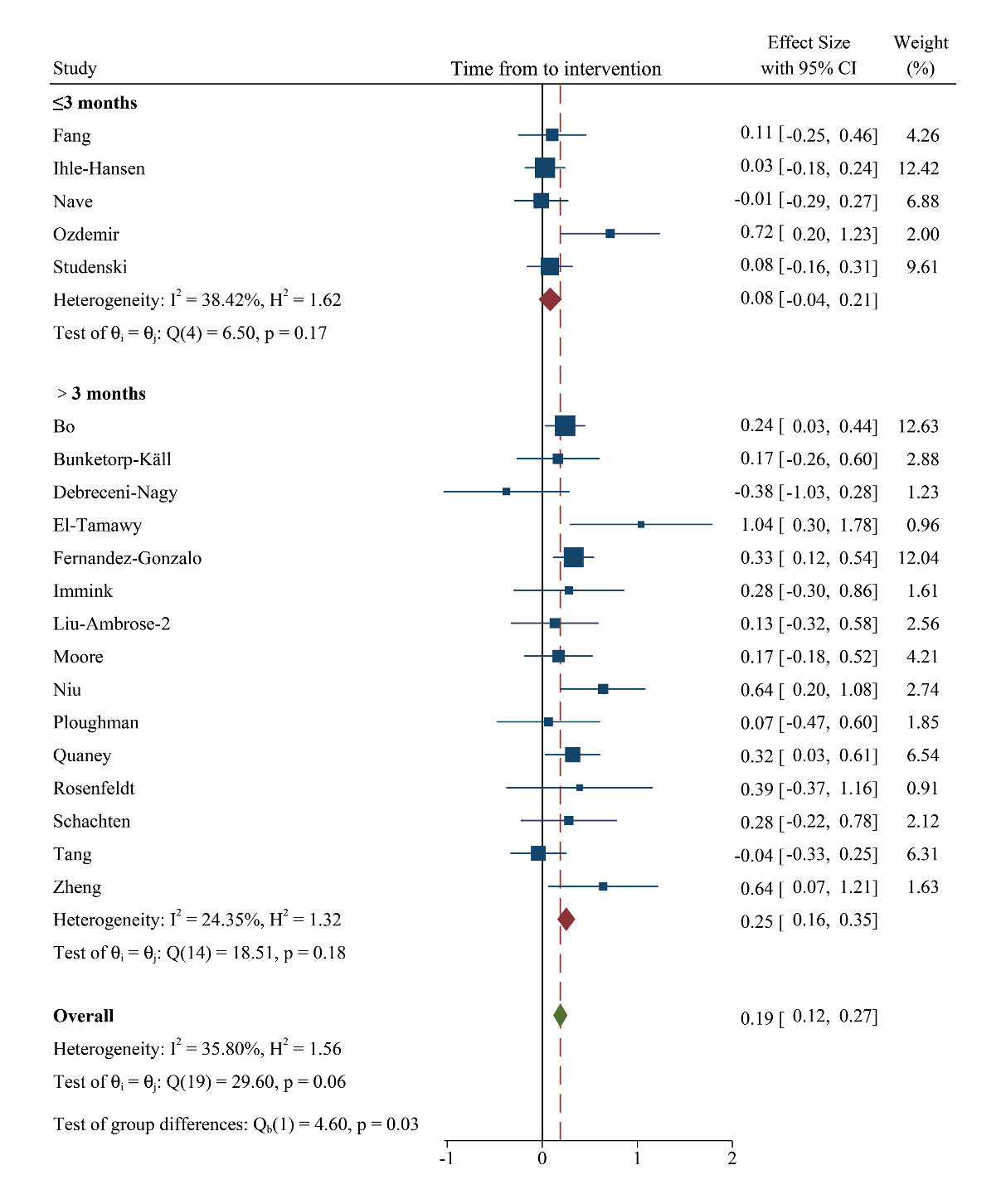
Abbreviation: CI, confidence interval; PA, physical activity.

**Supplementary Figure 5 Forest plot in the effect of cognitive status before intervention on the cognitive gains by PA.** Positive values of pooled effect size represented that PA improved cognitive performance.


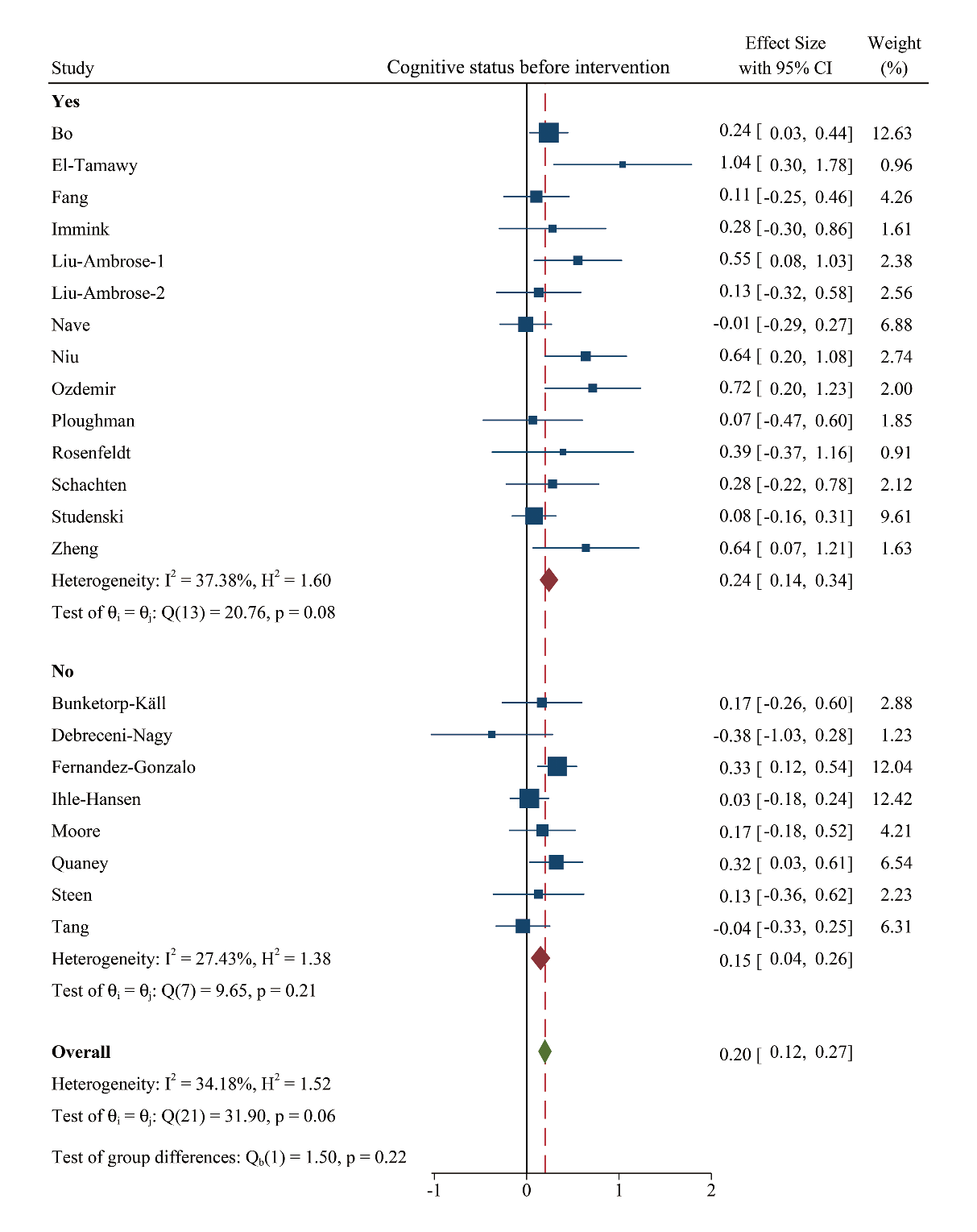
Abbreviation: CI, confidence interval; PA, physical activity.

**Supplementary Figure 6 Forest plot in the effect of type of measurement on the cognitive gains by PA.** Positive values of pooled effect size represented that PA improved cognitive performance.


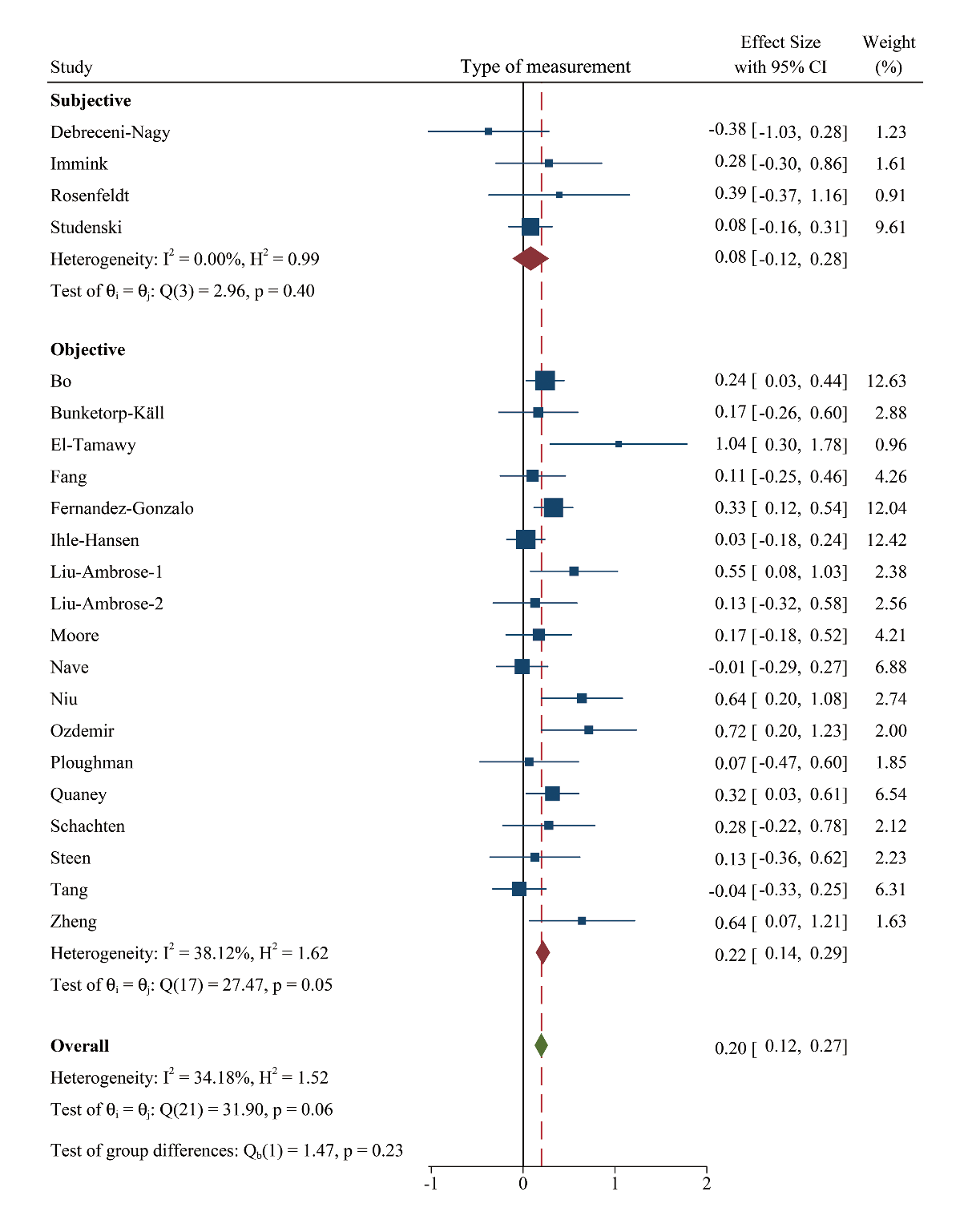
Abbreviation: CI, confidence interval; PA, physical activity.

**Supplementary Figure 7 Forest plot in the effect of type of PA on the cognitive gains by PA.** Positive values of pooled effect size represented that PA improved cognitive performance.


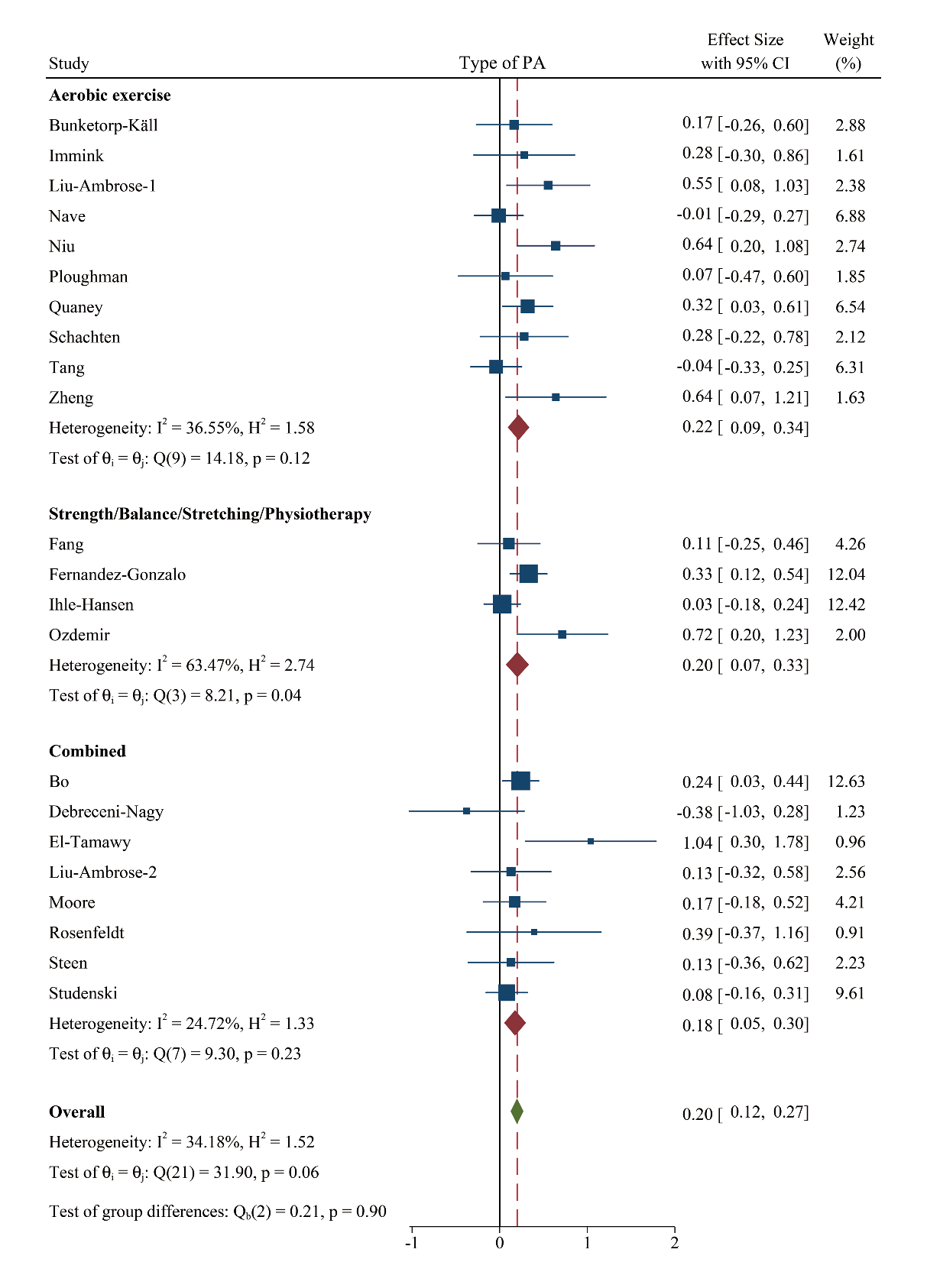
Abbreviation: CI, confidence interval; PA, physical activity.

**Supplementary Figure 8 Forest plot in the effect of PA intensity on the cognitive gains by PA.** Positive values of pooled effect size represented that PA improved cognitive performance.


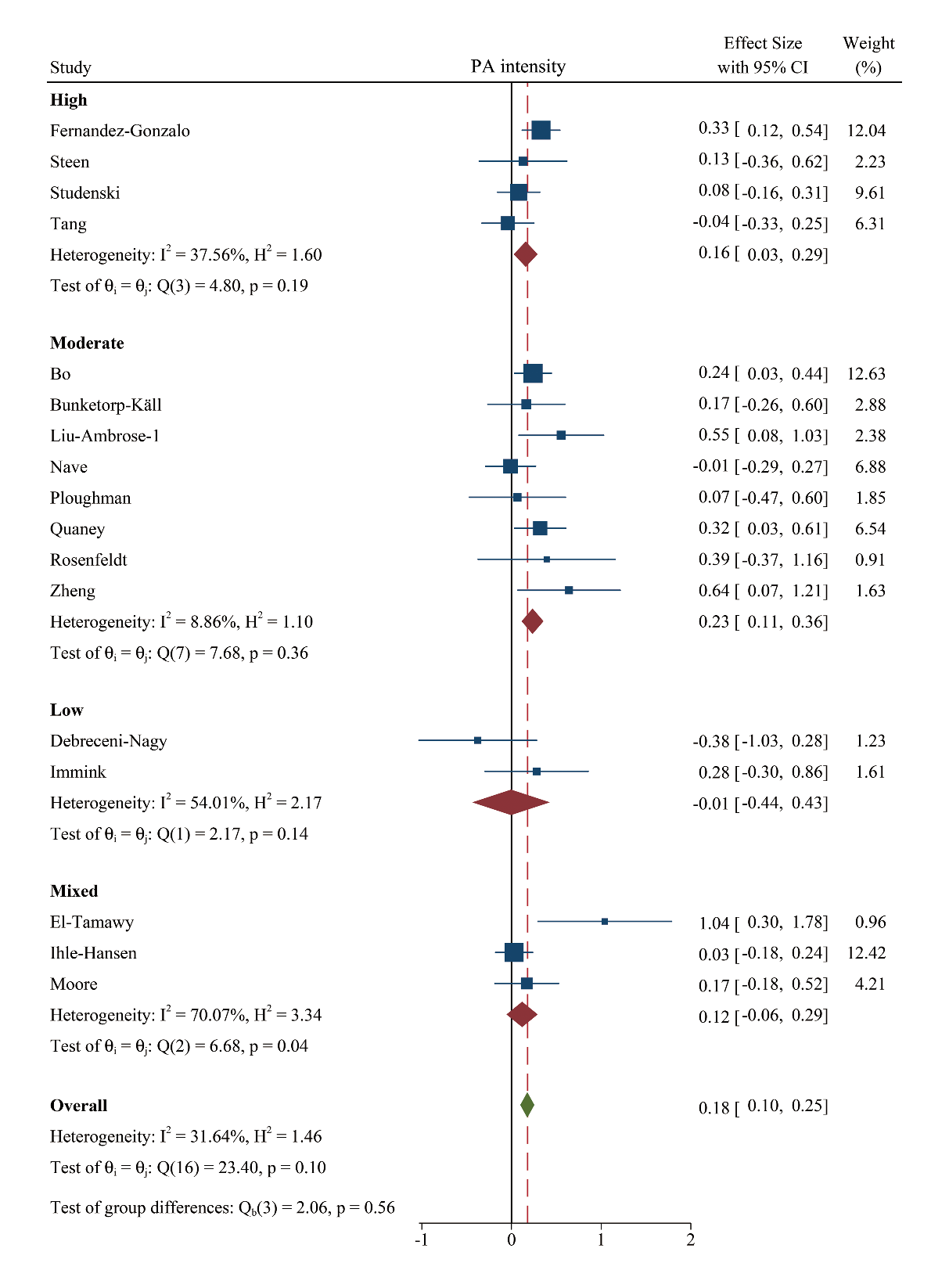
Abbreviation: CI, confidence interval; PA, physical activity.

**Supplementary Figure 9 Forest plot in the effect of duration of PA on the cognitive gains by PA.** Positive values of pooled effect size represented that PA improved cognitive performance.


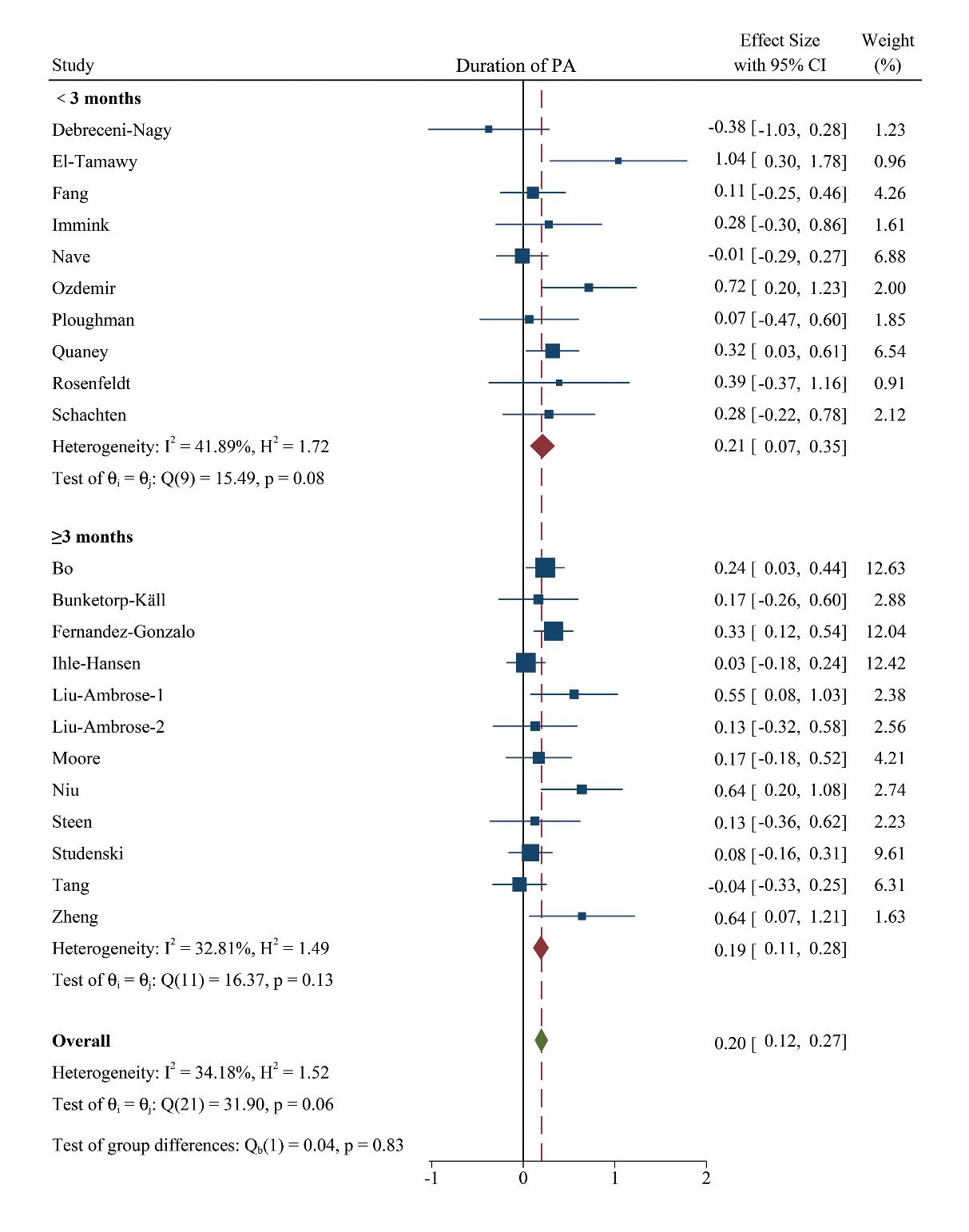
Abbreviation: CI, confidence interval; PA, physical activity.

**Supplementary Figure 10 Forest plot in the effect of type of aerobic exercise on the cognitive gains by PA.** Positive values of pooled effect size represented that PA improved cognitive performance.


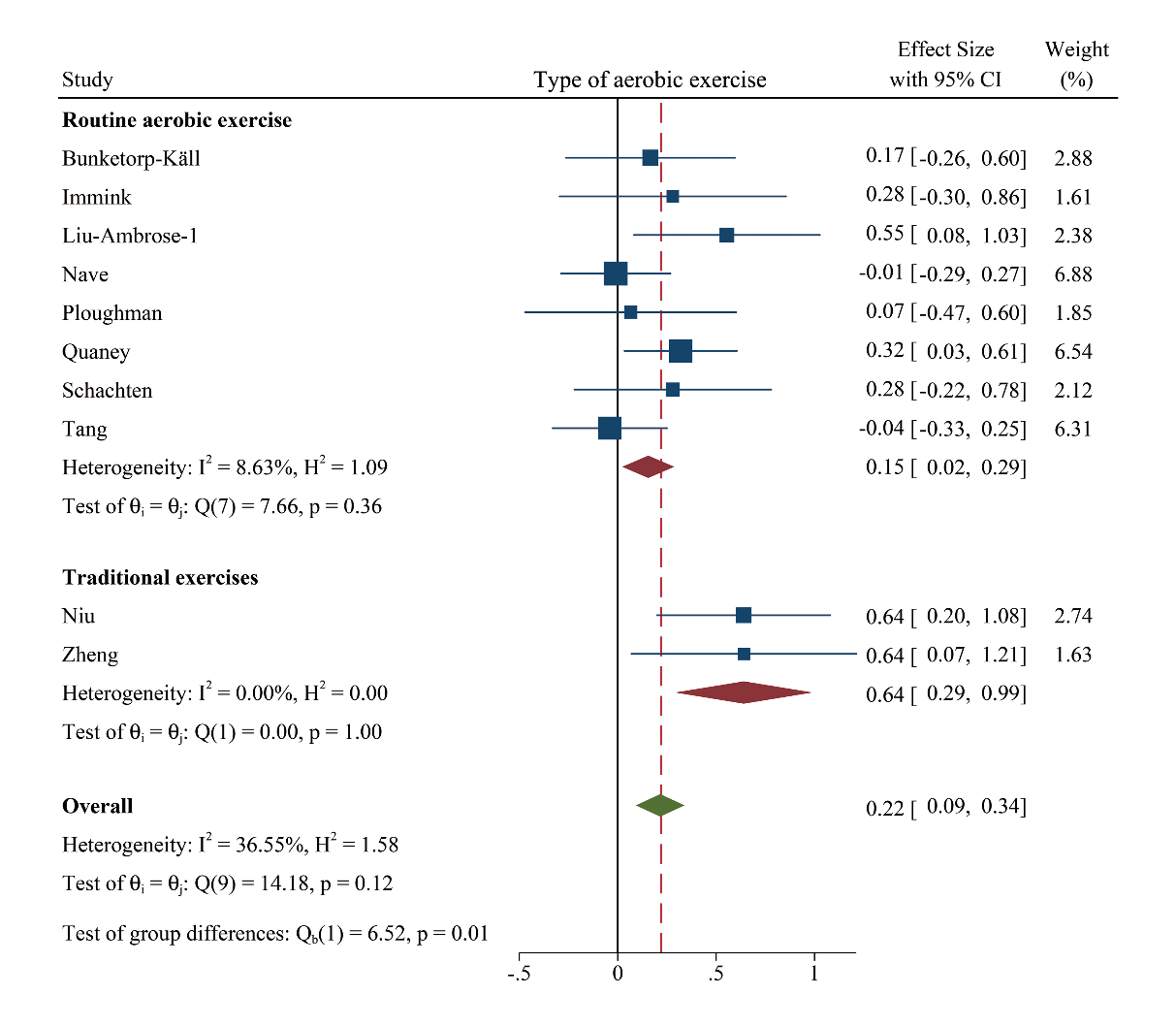
Abbreviation: CI, confidence interval; PA, physical activity.

**Supplementary Figure 11 Forest plot in the effect of type of control on the cognitive gains by PA.** Positive values of pooled effect size represented that PA improved cognitive performance.


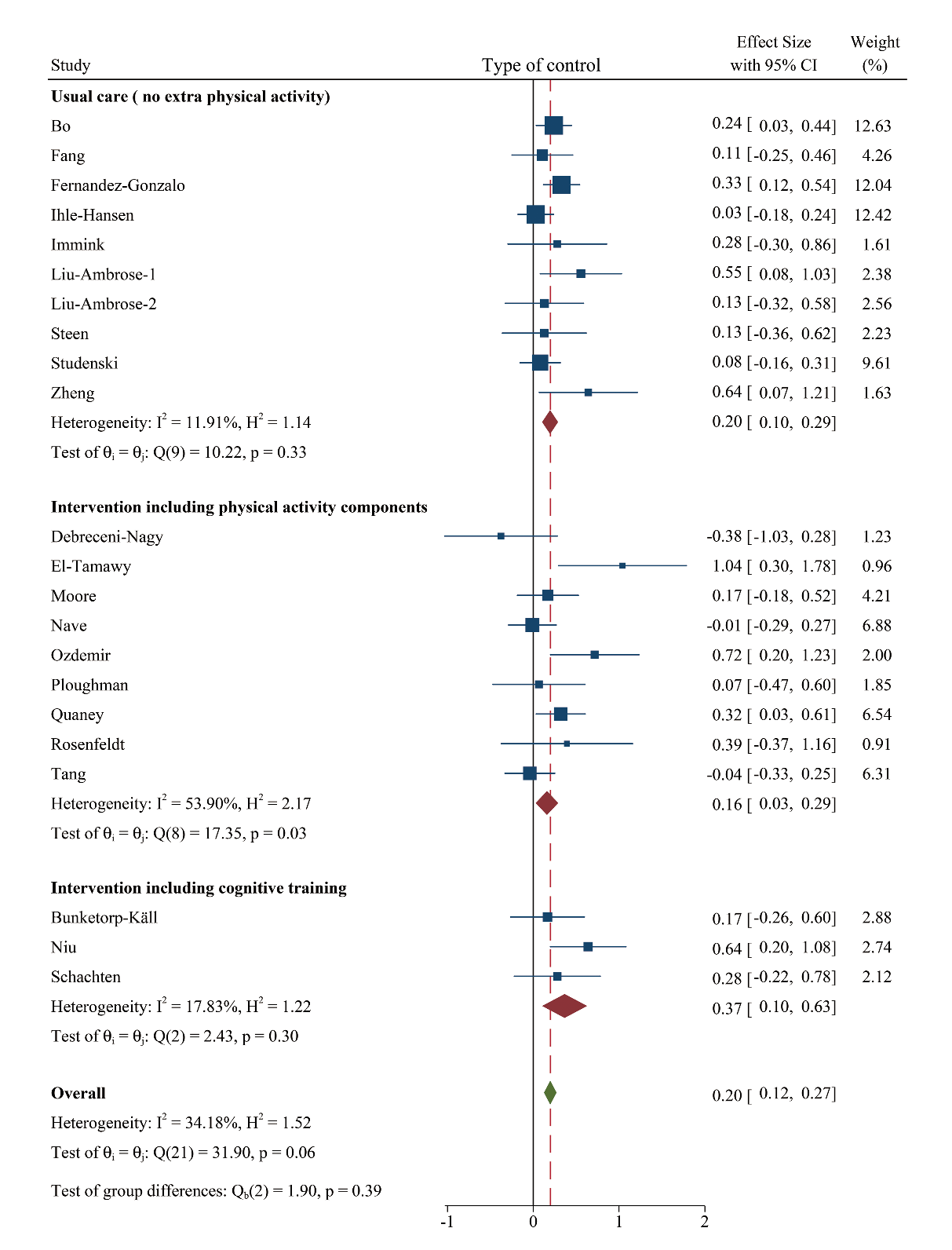
Abbreviation: CI, confidence interval; PA, physical activity
